# Retinoids rescue ceruloplasmin secretion and alleviate oxidative stress in Wilson’s disease-specific hepatocytes

**DOI:** 10.1093/hmg/ddac080

**Published:** 2022-04-07

**Authors:** Dan Song, Gou Takahashi, Yun-Wen Zheng, Mami Matsuo-Takasaki, Jingyue Li, Miho Takami, Yuri An, Yasuko Hemmi, Natsumi Miharada, Tsuyoshi Fujioka, Michiya Noguchi, Takashi Nakajima, Megumu K Saito, Yukio Nakamura, Tatsuya Oda, Yuichiro Miyaoka, Yohei Hayashi

**Affiliations:** iPS Cell Advanced Characterization and Development Team, RIKEN BioResource Research Center, Ibaraki 305-0074, Japan; Department of Gastrointestinal and Hepato-Biliary-Pancreatic Surgery, Faculty of Medicine, University of Tsukuba, Ibaraki 305-8575, Japan; Regenerative Medicine Project, Tokyo Metropolitan Institute of Medical Science, Tokyo 156-8506, Japan; Guangdong Provincial Key Laboratory of Large Animal Models for Biomedicine, and School of Biotechnology and Heath Sciences, Wuyi University, Guangdong 529020, China; Department of Medicinal and Life Sciences, Faculty of Pharmaceutical Sciences, Tokyo University of Science, Chiba 278-8510, Japan; Department of Regenerative Medicine, Graduate School of Medicine, Yokohama City University, Kanagawa 234-0006, Japan; Division of Regenerative Medicine, Center for Stem Cell Biology and Regenerative Medicine, Institute of Medical Science, The University of Tokyo, Tokyo 108-8639, Japan; iPS Cell Advanced Characterization and Development Team, RIKEN BioResource Research Center, Ibaraki 305-0074, Japan; iPS Cell Advanced Characterization and Development Team, RIKEN BioResource Research Center, Ibaraki 305-0074, Japan; iPS Cell Advanced Characterization and Development Team, RIKEN BioResource Research Center, Ibaraki 305-0074, Japan; iPS Cell Advanced Characterization and Development Team, RIKEN BioResource Research Center, Ibaraki 305-0074, Japan; iPS Cell Advanced Characterization and Development Team, RIKEN BioResource Research Center, Ibaraki 305-0074, Japan; Cell Engineering Division, BioResource Research Center, RIKEN, Ibaraki 305-0074, Japan; Cell Engineering Division, BioResource Research Center, RIKEN, Ibaraki 305-0074, Japan; Cell Engineering Division, BioResource Research Center, RIKEN, Ibaraki 305-0074, Japan; National Hospital Organization, Niigata National Hospital, Niigata 945-8585 Japan; Department of Clinical Application, Center for iPS Cell Research and Application (CiRA), Kyoto University, Kyoto 606-8507, Japan; Cell Engineering Division, BioResource Research Center, RIKEN, Ibaraki 305-0074, Japan; Department of Gastrointestinal and Hepato-Biliary-Pancreatic Surgery, Faculty of Medicine, University of Tsukuba, Ibaraki 305-8575, Japan; Regenerative Medicine Project, Tokyo Metropolitan Institute of Medical Science, Tokyo 156-8506, Japan; Graduate School of Medical and Dental Sciences, Tokyo Medical and Dental University, Tokyo 113-8510, Japan; Graduate School of Humanities and Sciences, Ochanomizu University, Tokyo 112-8610, Japan; iPS Cell Advanced Characterization and Development Team, RIKEN BioResource Research Center, Ibaraki 305-0074, Japan

## Abstract

Wilson’s disease (WD) is a copper metabolic disorder caused by a defective ATP7B function. Conventional therapies cause severe side effects and significant variation in efficacy, according to cohort studies. Thus, exploring new therapeutic approaches to prevent progression to liver failure is urgent. To study the physiology and pathology of WD, immortalized cell lines and rodent WD models have been used conventionally; however, a large gap remains among different species as well as in genetic backgrounds among individuals. We generated induced pluripotent stem cells (iPSCs) from four WD patients carrying compound heterozygous mutations in the *ATP7B* gene. ATP7B loss- and gain-of-functions were further manifested with ATP7B-deficient iPSCs and heterozygously corrected R778L WD patient-derived iPSCs using CRISPR-Cas9-based gene editing. Although the expression of ATP7B protein varied among WD-specific hepatocytes differentiated from these iPSCs, the expression and secretion of ceruloplasmin (Cp), a downstream copper carrier in plasma, were consistently decreased in WD patient-derived and ATP7B-deficient hepatocytes. A transcriptome analysis detected abnormalities in the retinoid signaling pathway and lipid metabolism in WD-specific hepatocytes. Drug screening using WD patient-derived hepatocytes identified retinoids as promising candidates for rescuing Cp secretion. All-trans retinoic acid also alleviates reactive oxygen species production induced by lipid accumulation in WD-specific hepatocytes treated with oleic acid. These patient-derived iPSC-based hepatic models function as effective platforms for the development of potential therapeutics for hepatic steatosis in WD and other fatty liver diseases.

## Introduction

Wilson’s disease (WD) (OMIM #277900), an autosomal recessive disorder, is majorly characterized by the accumulation of copper in the liver, leading to a series of metabolic disorders in the liver and nervous systems (1). WD is caused by a defective *ATP7B* gene, which is located on chromosome 13 and contains 21 exons coding a protein of 1465 amino acids, known as copper-transporting P-type ATPase. More than 600 disease-causing mutations that span almost all exons have been reported on the *ATP7B* gene (2,3), and the patterns of specific mutations are differently distributed among ethnic groups (4). The estimated incidence of WD is about 1:30 000 (1,4), whereas the carrier frequency is up to 1 in 90. WD patients require lifelong medical treatment. Conventional therapies, such as penicillamine, trientine, zinc salt, and tetrathiomolybdate, cause severe side effects and show significant variation in efficacy according to cohort studies (5–8). Thus, the identification of new therapeutic approaches to prevent aggravation and progression is urgent (9,10).

To study the physiology and pathology of WD, immortalized cell lines (11,12) and rodent WD models (13–16) have been used conventionally; however, a large gap remains among species as well as among genetic backgrounds in individuals. Patient-derived induced pluripotent stem cells (iPSCs) enable the generation of human hepatocytes to study WD physiology and pathology (17–24). Previous studies have majorly focused on high-frequency hot spot mutations in WD patients (25). However, due to the limited numbers of patients included in these studies, which prevented accurate assessments of significance, the generally targetable features of WD caused by variable mutations have not been fully identified.

In this study, we generated iPSC lines from four patients carrying compound mutations on the *ATP7B* gene. In addition, we generated two ATP7B-deficient iPSC lines via deletion-mutagenesis and point-mutagenesis from a healthy-donor (HD) iPSC line and mutation-corrected iPSCs from R778L homozygous mutant iPSCs generated previously by gene editing, respectively. (17–20). Using these WD-specific and ATP7B-deficient iPSC lines, we explored the disease phenotype–genotype correlation and searched for novel therapeutic candidates. We focused on ceruloplasmin (Cp), a copper-containing plasma ferroxidase synthesized in hepatocytes and secreted into plasma following the incorporation of six atoms of copper by ATP7B protein in the trans-Golgi network (26). A low concentration of serum Cp is generally used as a diagnostic criterion of WD (27,28). We successfully recapitulated the reduction of Cp expression and secretion in WD-iPSC-derived hepatocytes. A transcriptome analysis identified differentially regulated genes in WD-specific hepatocytes that led to abnormalities in the retinoid signaling pathways and lipid metabolism. In small drug screening to restore Cp secretion in WD-iPSC-derived hepatocytes, we identified all-trans retinoic acid (ATRA) and clinically approved retinoids as promising candidates. In addition, ATRA alleviated reactive oxygen species (ROS) production induced by lipid accumulation in oleic acid-treated WD-specific hepatocytes. Since Cp reduction and liver steatosis are the initial symptoms of WD, our results suggest that retinoids may be able to prevent or delay the progression of these symptoms.

## Results

### Generation and characterization of patient-derived WD-iPSCs

**Figure 1:**
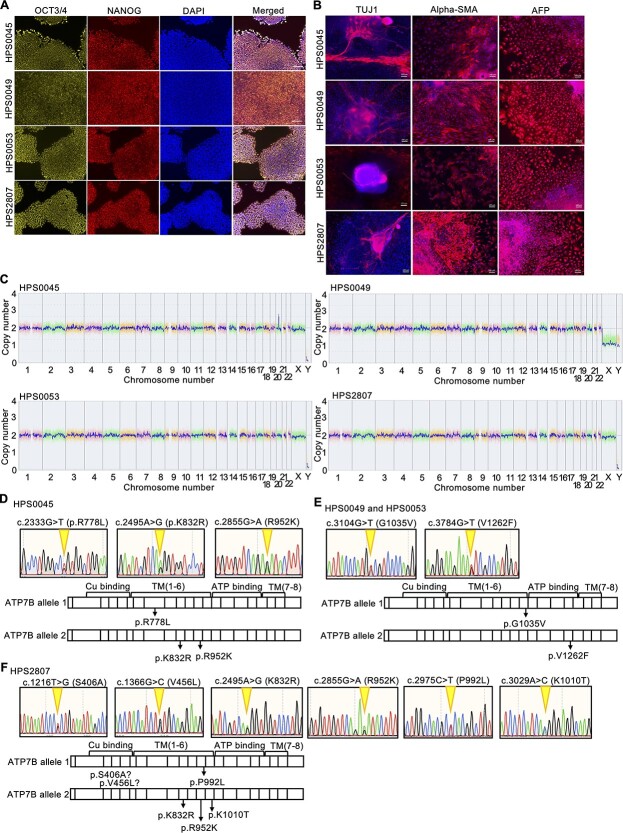
Generation of WD patient-derived iPSC lines carrying compound heterozygous *ATP7B* mutations. (**A**) Expression of self-renewal markers of iPSCs, OCT3/4 (yellow) and NANOG (red), in WD patient-derived iPSCs. DAPI was used to stain nuclei (blue). Scale bar = 100 μm. (**B**) Pluripotency in WD patient-derived iPSCs was evaluated with an EB formation assay. TUJ1 (ectoderm marker)/alpha-SMA (mesoderm marker)/AFP (endoderm marker) expression of EB. Scale bar = 100 μm. (**C**) Genome-wide copy number variation detection using a CGH array analysis for the WD patient-derived iPSC lines established in this study. (**D–F**) Sequence results and schematic illustration of the allelic mutation positions of *ATP7B* gDNA in these WD patient-derived iPSC lines, HPS0045 in (D), HPS0049, and HPS0053 in (E) and HPS2807 in (F), generated in this study.

We generated patient-derived iPSC lines from four patients who suffered from WD (HPS0045, HPS0049, HPS0053 and HPS2807) using retroviral vectors or nonintegrating episomal DNA vectors (Supplementary Material, Table S1). All four WD-iPSC lines highly expressed self-renewal markers of iPSCs, such as OCT3/4, NANOG, SOX2 and KLF4 (Fig. 1A and Supplementary Material, Fig S1A). An embryoid body (EB) formation assay, which allowed the spontaneous differentiation of iPSCs into three germ layers *in vitro*, revealed the pluripotency of all four lines (Fig. 1B). In addition, teratomas containing various tissues derived from the three germ layers were formed from these WD-iPSC lines (Supplementary Material, Fig. S1B). A comparative genome hybridization (CGH) array analysis showed that all of these WD-iPSC lines had a normal karyotype without large copy number variations (Fig. 1C). As *ATP7B* is the gene responsible for WD (29,30), we detected mutations in this gene carried in these WD-iPSC lines by sequencing the entire coding region of ATP7B from cDNA in WD-iPSC-derived hepatocyte cultures. We further verified the results with allelic identification partially in the cDNA and genomic DNA (gDNA) of these WD-iPSCs. Sequence results showed that the HPS0045 iPSC line carried R778L (c.2333G > T), K832R (c.2495A > G), and R952K (c.2855G > A) heterozygous mutations. Both the HPS0049 and HPS0053 iPSC lines carried G1035V (c.3104G > T) and V1262F (c.3784G > T) heterozygous mutations, because their donors were siblings. The HPS2807 iPSC line carried S406A (c.1216 T > G), V456L (c.1366G > C), K832R (c.2495A > G), R952K (c.2855G > A), P992L (c.2975C > T) and K1010T (c.3029A > C) heterozygous mutations (Fig. 1D–F). These characterization results indicated that these WD-iPSC lines maintained self-renewal, pluripotency, genomic integrity and compound heterozygous mutations in the *ATP7B* gene.

### Hepatic differentiation of WD-iPSC lines

To establish disease models of WD *in vitro*, WD-iPSC lines and an iPSC line from a healthy donor [i.e. WTC11 line (GM25256)] were differentiated into hepatocytes using a monolayer differentiation protocol (Supplementary Material, Fig. S2A) that was modified from a protocol reported in a previous study (31). In our differentiation protocol, cells highly expressed the definitive endodermal marker genes *SOX17*, *HNF4A* and *DLK1* on days 4–7 (Supplementary Material, Fig. S2B) and the hepatoblast and/or hepatocyte marker genes alpha-fetoprotein (*AFP*), albumin (*ALB*) and alpha-1-antitrypsin (*A1AT)* on day 17 (Supplementary Material, Fig. S2C). Immunostaining showed that the definitive endodermal marker proteins SOX17 and GATA6 were highly expressed on day 5 (Supplementary Material, Fig. S2D and E) and that the hepatic marker proteins HNF4A, AFP and ALB were highly expressed on day 17 (Supplementary Material, Fig. S2F–H). We also detected the expression of ATP7B protein on day 17 but not in undifferentiated iPSCs (Supplementary Material, Fig. S2I). WD-specific hepatocytes also showed a comparable expression of hepatic marker genes and proteins to WTC11-Hep (hepatocytes differentiated from WTC11 iPSC line) on differentiation day 17 (Supplementary Material, Fig. S3A–D). These results show that the ability of WD-specific iPSCs and WTC11 iPSCs to differentiate into hepatic lineages is comparable, thus supporting the suitability of our present hepatic differentiation protocol for WD modeling *in vitro*.

### Cp expression is consistently decreased in WD-iPSC-derived hepatocytes

**Figure 2:**
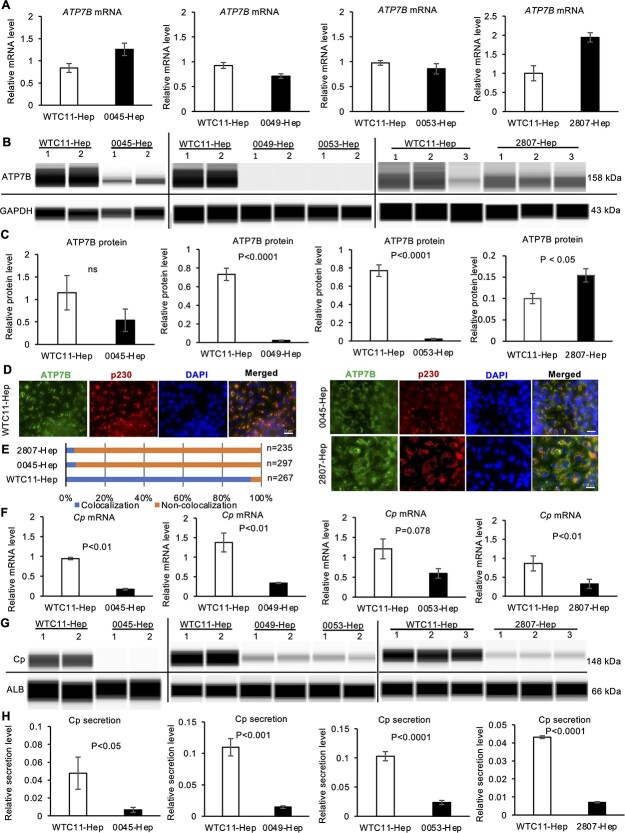
The expression and secretion of Cp are consistently decreased in WD-iPSC-derived hepatocytes. (**A**) Expression of *ATP7B* mRNA in WD-iPSC-derived hepatocytes on differentiation day 17 quantified by RT-qPCR (0045-Hep, *n* = 3; 0053-Hep, *n* = 3; 0049-Hep, *n* = 4; 2807-Hep, *n* = 3). Data are shown as the mean ± SEM. (**B**) Representative images of western blot results of ATP7B protein expression in WTC11- and WD-iPSC-derived hepatocytes (WTC11-Hep, 0045-Hep, 0049-Hep and 0053-Hep, respectively) on differentiation day 17. GAPDH protein was used as a housekeeping control. (**C**) Bar graphs showing the quantified expression of ATP7B protein normalized with the expression of GAPDH protein. The data obtained from WTC11-Hep were used as the common control for each WD-Hep (0045-Hep, *n* = 4; 0053-Hep, *n* = 6; 0049-Hep, *n* = 7; 2807-Hep, *n* = 3). Data are shown as the mean ± SEM. *P*-values were determined by an unpaired two-tailed Student’s *t*-test. (**D**) Immunocytochemistry of ATP7B and p230 trans-Golgi in WD-iPSC-derived hepatocytes. Representative images costained with DAPI are shown. Scale bar = 25 μm. (**E**) Quantification of cells showing colocalization of ATP7B and p230. Counted cell numbers (*n*) are shown from representative experiments. (**F**) Expression of *Cp* mRNA in WTC11-Hep and WD-iPSC-derived-Hep. Data are shown as the mean ± SEM. *P*-values were determined by an unpaired two-tailed Student’s *t*-test. (**G**) Representative images of western blot data of secreted Cp and ALB proteins by WTC11- and WD-iPSC-derived hepatocyte cultures on differentiation day 17. (**H**) Quantified data of Cp protein secretion after normalization with ALB secretion. WTC11-Hep was used as the common control for each WD-Hep (0045-Hep, *n* = 10; 0053-Hep, *n* = 7; WD0049-Hep, *n* = 6; 2807-Hep, *n* = 6). Data are shown as the mean ± SEM. *P*-values were determined by an unpaired two-tailed Student’s *t*-test.

We next explored how WD mutations affected the ATP7B mRNA and protein expression and localization in WD-iPSC-derived hepatocytes. In our differentiation protocol, the *ATP7B* mRNA expression increased by roughly 7-fold by differentiation day 17 (Supplementary Material, Fig. S3E). The expression of ATP7B mRNA and protein was therefore examined in WD-specific hepatocytes on differentiation day 17 using quantitative reverse transcription polymerase chain reaction (RT-qPCR) and western blotting, respectively. The expression of mRNA in HPS0045- and HPS2807-derived hepatocytes was elevated by 1.5- or 2-fold compared with WTC11-derived hepatocytes, whereas the expression in HPS0049- and HPS0053-derived hepatocytes was slightly lower than or not significantly different from that in WTC11-derived hepatocytes (Fig. 2A). The expression of ATP7B protein in HPS0045-derived hepatocytes decreased by around half (not significant) to that in WTC11-derived hepatocytes, and HPS2807-derived hepatocytes expressed a significantly higher amount of ATP7B protein than WTC11-derived hepatocytes, whereas HPS0049- and HPS0053-derived hepatocytes expressed no or undetectable levels of ATP7B protein (Fig. 2B and C). We next examined the protein localization of ATP7B in HPS2807- and HPS0045-derived hepatocytes. Although almost all the ATP7B protein in WTC11-derived hepatocytes was co-localized with p230, a marker of the trans-Golgi network, a considerable portion of ATP7B protein in most HPS0045- and HPS2807-derived hepatocytes leaked out from the trans-Golgi network (Fig. 2D and E). These results suggested that WD-specific iPSC-derived hepatocytes expressed either or both decreased levels of ATP7B protein and/or its mislocalization, which might be regulated at the posttranscriptional level, depending on the type of ATP7B mutation involved.

Next, we examined the mRNA expression and secretion of Cp, which receives copper ions from the ATP7B protein in hepatocytes and is then secreted into the plasma to deliver these ions throughout the body. The expression of Cp was markedly increased during differentiation into hepatocytes from undifferentiated iPSCs (Supplementary Material, Fig. S3F). In iPSC-derived hepatocytes on differentiation day 17, the expression of Cp mRNA was generally lower in WD-specific hepatocytes than in WTC11-derived hepatocytes (Fig. 2F). In addition, the degree of Cp secretion, which was normalized by the amount of ALB secreted to evaluate the Cp secretion per hepatocyte, was generally lower in WD-specific hepatocytes than in WTC11-derived hepatocytes (Fig. 2G and H). These results suggested that WD mutations in *ATP7B* generally resulted in reduced mRNA expression and protein secretion of Cp in iPSC-derived hepatocytes.

### Gene-editing of ATP7B in iPSC-derived hepatocytes confirmed the regulation of Cp expression and secretion by ATP7B

**Figure 3:**
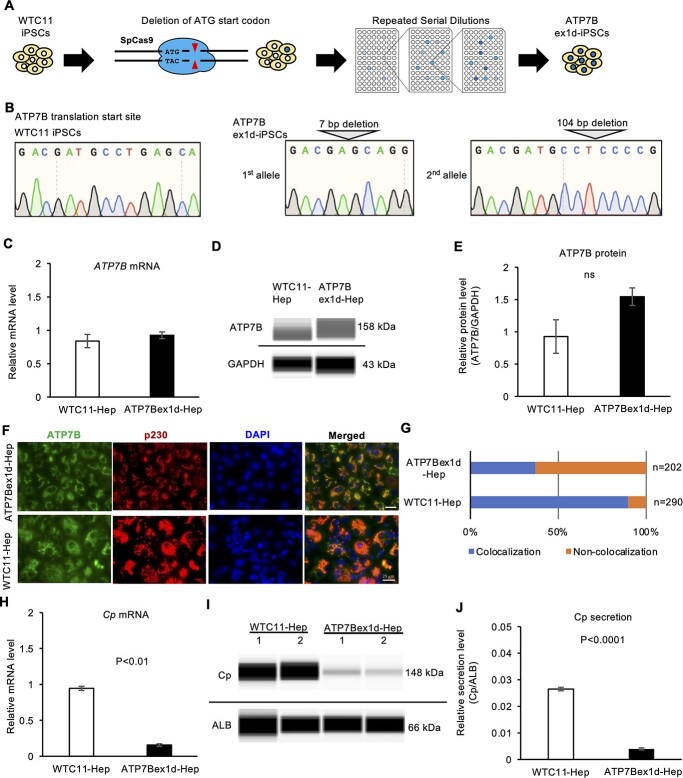
The expression and secretion of Cp are decreased in *ATP7B*-exon1-deletion-iPSC-derived hepatocytes. (**A**) Schematic illustration of the generation of *ATP7B*-exon1-deletion (ex1d) iPSCs using gene-editing technology. (**B**) Allele-specific sequence data around the start codon of *ATP7B* gDNA in a gene-edited iPSC clone. (**C**) The expression of *ATP7B* mRNA in WTC11-Hepatocytes and ATP7B-ex1d-Hepatocytes. Data are shown as the mean ± SEM (*n* = 3). (**D**) Representative image of western blot data of ATP7B and GAPDH proteins from parental WTC11- and ATP7B-ex1d-hepatocyte cultures on differentiation day 17. (**E**) Quantified data of ATP7B protein expression after normalization with GAPDH protein. Data are shown as the mean ± SEM (*n* = 3). (**F**) Immunocytochemistry of ATP7B and p230 trans-Golgi in ATP7B-ex1d-iPSC-derived hepatocytes. Representative images costained with DAPI are shown. Scale bar = 25 μm. (**G**) Quantification of cells showing colocalization of ATP7B and p230. Counted cell numbers (*n*) are shown from representative experiments. (**H**) The expression of *Cp* mRNA in WTC11-Hep and ATP7B-ex1d-Hep. Data are shown as the mean ± SEM (*n* = 3). *P*-values were determined by an unpaired two-tailed Student’s *t*-test. (**I**) Representative image of western blot data of Cp and ALB proteins secreted by WTC11- and ATP7B-ex1d-Hep on differentiation day 17. (**J**) Quantified data of secreted Cp protein after normalization with ALB protein in WTC11- and ATP7B-ex1d-Hep. Data are shown as the mean ± SEM (*n* = 3). *P*-values were determined by an unpaired two-tailed Student’s *t*-test.

To verify the effect of loss-of-function of *ATP7B* with isogenic backgrounds in iPSC-derived hepatocyte models, we generated an iPSC clone carrying *ATP7B* exon 1 deletion from the WTC11 iPSC line using the CRISPR-Cas9 system (32). The translational start codon of the *ATP7B* gene was targeted by a guide RNA (gRNA) and Cas9 in the WTC11 iPSC line (Fig. 3A). We isolated iPSC clones in which 7 bp had been deleted from *ATP7B* exon 1 in one allele and 104 bp spanning exon 1 and intron 1 had been deleted from the other allele, as detected by genotyping sequencing (Fig. 3B). The expression of hepatic markers was comparable between this ATP7B exon 1-deletion (ex1d) iPSC clone and the original WTC11-derived hepatocytes on differentiation day 17 (Supplementary Material, Fig. S4A–G). The *ATP7B* gene and protein expression were examined in these ATP7B-ex1d hepatocytes with RT-qPCR and western blotting, respectively. These ATP7B-ex1d hepatocytes showed comparable *ATP7B* gene and protein expression to WTC11-derived hepatocytes (Fig. 3C–E). In contrast, many ATP7B-ex1d hepatocytes carried mislocalized ATP7B protein from the trans-Golgi network (Fig. 3F and G). The mRNA expression and secretion of Cp were significantly lower in ATP7B-ex1d hepatocytes than the original line-derived hepatocyte (Fig. 3H–J). These results indicated that the deletion in ATP7B exon 1 results in producing the defective ATP7B protein from alternative start codons because there were several ATG sites in exon 2 and caused reduced mRNA expression and protein secretion of Cp in iPSC-derived hepatocytes.

**Figure 4:**
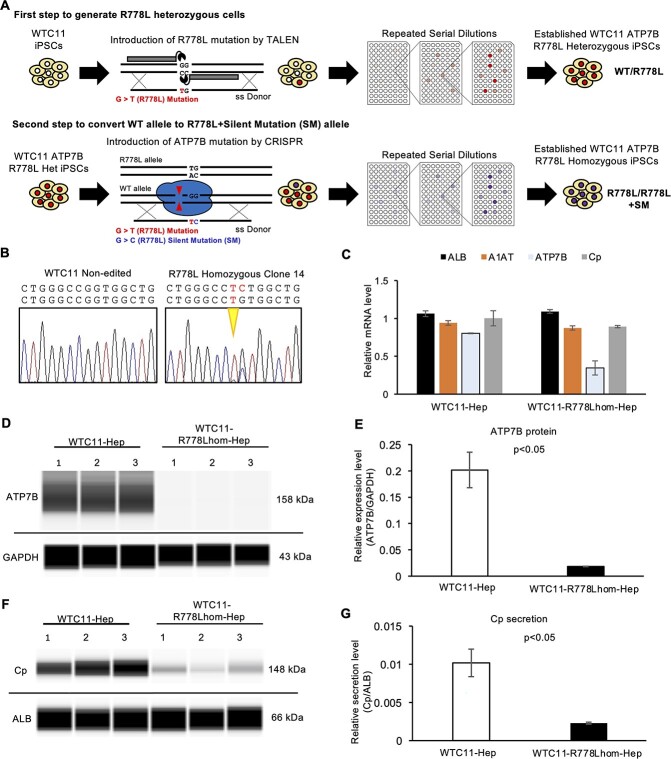
ATP7B protein and Cp secretion are decreased in WTC11-R778Lhom-iPSC-derived hepatocytes. (**A**) Schematic illustration of the generation of WTC11-R778Lhom-iPSC by gene-editing technology. (**B**) Sequence results around the R778L position of *ATP7B* gDNA in WTC11 and the established WTC11-R778Lhom-iPSC clone. (**C**) The expression of *ALB*, *A1AT*, *ATP7B* and *Cp* mRNA in WTC11-Hep and WTC11-R778Lhom-Hep. Data are shown as the mean ± SEM (*n* = 3). (**D**) Representative image of western blot data of ATP7B and GAPDH proteins from WTC11- and WTC11-R778Lhom-derived hepatocyte cultures on differentiation day 16. (**E**) Quantified data of ATP7B protein expression after normalization with GAPDH protein. Data are shown as the mean ± SEM (*n* = 3). *P*-values were determined by an unpaired two-tailed Student’s *t*-test. (**F**) Representative image of western blot data of secreted Cp and ALB proteins by WTC11-Hep and WTC11-R778Lhom-Hep on differentiation day 16. (**G**) Quantified data of secreted Cp protein after normalization with ALB protein in WTC11-Hep and WTC11-R778Lhom-Hep. Data are shown as the mean ± SEM (*n* = 3). *P*-values were determined by an unpaired two-tailed Student’s *t*-test.

To further verify the effect of *ATP7B* mutations directly, we generated ATP7B R778L homozygous (R778Lhom-) iPSCs from WTC11 iPSCs. First, an R778L mutation was introduced into one allele by TALEN to establish the WTC11 ATP7B R778L heterozygous (R778Lhet-) iPSCs. The R778L + silent mutation (SM) allele was then introduced into the wild-type (WT) allele of WTC11 ATP7B R778L heterozygous iPSCs using the CRISPR-Cas9 system (Fig. 4A). As shown in the sequence results of the resulting cell line, one allele carried the R778L mutation, whereas the other carried the R778L mutation plus one SM in the newly established line (Fig. 4B). The expression of hepatic markers was examined by RT-qPCR, which showed comparable levels of hepatocyte gene expression (ALB and A1AT) between WTC11-R778Lhom-iPSCs and original WTC11-derived hepatocytes (Fig. 4C). The expression of both ATP7B mRNA and protein was decreased in the WTC11-R778Lhom-derived hepatocytes (Fig. 4C–E). Although the WTC11-R778Lhom-Hep showed a comparable expression of Cp mRNA to the original WTC11-derived hepatocytes, their Cp secretion capacity was severely compromised (Fig. 4C, F and G). Our results suggested that the R778L homozygous mutation disrupted the ATP7B expression at both the transcriptional and posttranscriptional levels, one possible reason for the compromised Cp secretion.

**Figure 5:**
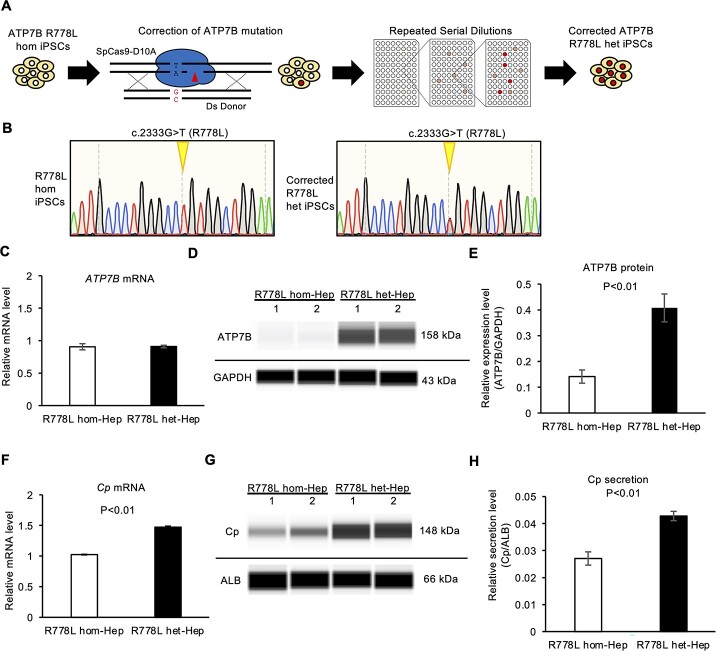
The expression and secretion of Cp are increased in heterozygously corrected ATP7B R778L*-*iPSC-derived hepatocytes. (**A**) Schematic illustration of the generation of heterozygously corrected *ATP7B* R778L iPSCs using gene-editing technology. (**B**) Sequence results around the R778L position of *ATP7B* gDNA in parental R778L homozygous iPSCs (R778Lhom) and the established gene-edited iPSC clone (R778Lhet). (**C**) The expression of *ATP7B* mRNA in WTC11-Hep and ATP7B-edited-Hep on differentiation day 17. Data are shown as the mean ± SEM (*n* = 3). (**D**) Representative image of western blot data of ATP7B and GAPDH proteins from parental R778Lhom- and R778Lhet-iPSC-derived hepatocytes on differentiation day 17. (**E**) Quantified data of ATP7B protein expression after normalization with GAPDH protein. Data are shown as the mean ± SEM (*n* = 3). (**F**) Expression of *Cp* mRNA in R778Lhom-Hep and R778Lhet-Hep detected by RT-qPCR. Data are shown as the mean ± SEM (*n* = 3). *P*-values were determined by an unpaired two-tailed Student’s *t*-test. (**G**) Representative image of western blot data of Cp and ALB proteins secreted by R778Lhom-Hep and R778Lhet-Hep on differentiation day 17. (**H**) Quantified data of secreted Cp protein after normalization with ALB protein in R778L hom-Hep and R778L het-Hep. Data are shown as the mean ± SEM (*n* = 3). *P*-values were determined by an unpaired two-tailed Student’s *t*-test.

To examine the effect of genetic correction of the ATP7B R778L mutation with isogenic backgrounds on WD disease modeling, we performed CRISPR-Cas9-based gene correction on one previously generated WD-iPSC line carrying the predominant homozygous mutation (R778L) (17) (Fig. 5A). We targeted the area near the R778L mutation site with gRNA using Cas9 D10A nickase, and an oligonucleotide DNA was used as a homologous recombination donor to generate scarlessly rescued iPSC subclones (33). After introducing this gRNA, double-stranded donor oligonucleotide DNA and Cas9 D10A nickase into R778L-homozygous iPSCs, we isolated single-cell clones and selected R778L-heterozygous iPSC subclones. The gDNA sequence of the exon in ATP7B gene was confirmed by Sanger sequencing (Fig. 5B). The hepatic differentiation capability of the corrected R778L-heterozygous iPSC subclone and the original R778L-homozygous iPSC line was examined by immunostaining and RT-qPCR. These two iPSC subclones expressed the comparable levels of hepatic markers (Supplementary Material, Fig. S5A–F). The *ATP7B* mRNA and protein levels were examined in these iPSC-derived hepatocytes. Although both iPSC clones expressed similar levels of *ATP7B* mRNA, the R778L-heterozygous line expressed a significantly higher level of ATP7B protein (Fig. 5C–E). Cp mRNA and secretion levels were further examined under these conditions. The R778L-heterozygous subclone expressed significantly higher levels of mRNA and secreted protein of Cp than did the original R778L-homozygous line (Fig. 5F–H). These results suggested that heterozygous correction of R778L rescued the Cp expression and secretion as well as the ATP7B protein expression and function. Taken together, our findings showed that the loss- and gain-of-function of ATP7B resulted in the regulation of Cp expression and secretion in iPSC-derived hepatocytes.

### A transcriptome analysis identified abnormalities of the RA signaling pathway and lipid metabolism in WD-specific hepatocytes

**Figure 6:**
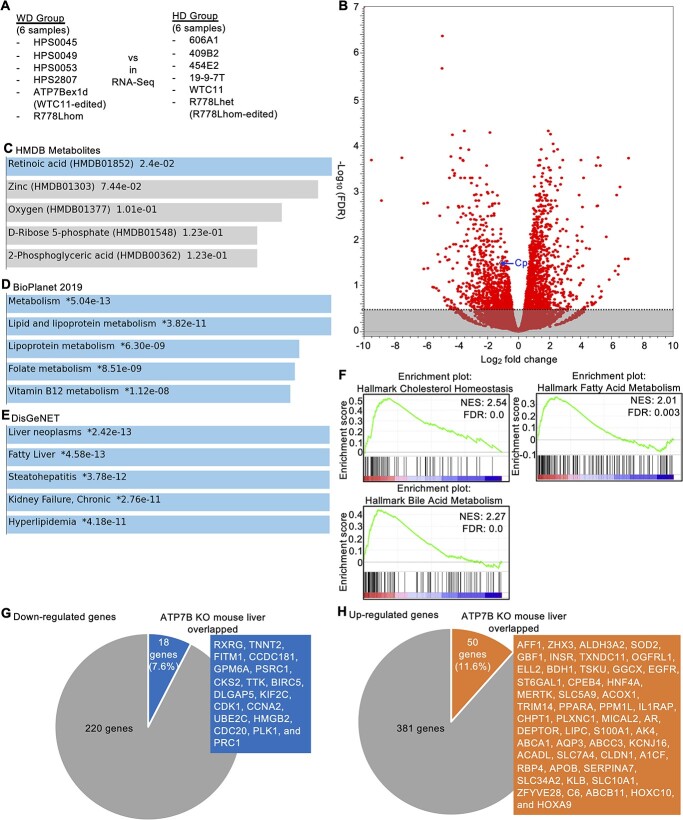
A transcriptome analysis showing abnormalities of the RA signal pathway and lipid metabolism in WD-iPSC-derived hepatocytes. (**A**) Schematic illustration of RNA-seq. (**B**) Volcano plot showing the Log_2_ fold change as the X-axis and −log_10_ (FDR) as the Y-axis. All genes are plotted in red circles. The Cp gene is indicated by a blue arrow. Nonsignificant genes with FDR ≥ 0.05 are masked in gray. (**C**) A bar chart showing candidate metabolites using the HMDB metabolites program for downregulated genes. Metabolite names (HMDB metabolite ID) and values of −log_10_(*P*-value) are shown. (**D**) A bar chart showing candidate pathways using the NCATS BioPlanet program for upregulated genes. Pathway names and values of −log_10_(*P*-value) are shown. (**E**) A bar chart showing candidate disease types using the DisGeNET program for upregulated genes. Disease names and values of −log_10_(*P*-value) are shown. (**F**) The findings concerning cholesterol homeostasis, fatty acid metabolism and bile acid metabolism in the GSEA are shown. The Y-axis represents the ES, and the X-axis shows the genes (vertical black lines) represented in gene sets. The green line connects points between the ES and genes. The ES is the maximum deviation from zero as calculated for each gene going down the ranked list and represents the degree of overrepresentation of a gene set at the top or bottom of the ranked gene list. The colored band at the bottom represents the degree of correlation of genes with the phenotype (red for positive and blue for negative correlation). The normalized enrichment score and false discovery ratio (FDR) are shown. (**G**) A pie chart showing overlapping downregulated genes in WD-specific hepatocytes and ATP7B KO mouse liver samples. The number of these genes, the percentage of overlapping genes and the overlapping gene name are shown. (**H**) A pie chart showing overlapping upregulated genes in WD-specific hepatocytes and ATP7B KO mouse liver samples. The number of these genes, the percentage of overlapping genes and the overlapping gene name are shown.

To identify the differences in the global gene expression patterns in WD-specific hepatocytes, we performed an RNA-seq analysis. Six WD-specific hepatocytes and six HD hepatocytes were compared in total (Fig. 6A). According to this comparison, 238 genes were significantly downregulated [false discovery rate (FDR) < 0.05)], and 431 were significantly upregulated (FDR < 0.05) in WD-specific hepatocytes (Fig. 6B and Supplementary Material, Table S2). Of note, Cp was included among these significantly downregulated genes. We then performed an enrichment analysis of the biological pathways and gene ontology on these differentially regulated genes. Regarding the downregulated genes, RA was the top metabolite enriched among the genes identified in the Human Metabolite DataBase (HMDB) program (Fig. 6C). Regarding the upregulated genes, lipid and lipoprotein metabolisms were the top pathways enriched among the genes identified in the National Center for Advancing Translational Sciences (NCATS) BioPlanet program (Fig. 6D). In addition, liver neoplasms, fatty liver and steatohepatitis were the top associated diseases enriched among the genes identified in the DisGeNET program (Fig. 6E). Furthermore, a gene set enrichment analysis (GSEA) was performed for the entire set of these whole transcriptome data. We found that genes related to cholesterol homeostasis, fatty acid metabolism and bile acid metabolism were positively enriched in the WD-specific hepatocytes group (Figure 6F and Supplementary Material, Table S3).

To examine these differentially regulated genes in an ATP7B-deficient context, we performed an integrated analysis of these genes with previous datasets from samples of mouse liver comparing ATP7B knockout (KO) mice with WT mice (*n* = 4). We identified genes that were significantly down- or upregulated in both our WD-specific hepatocytes and a dataset of ATP7B KO mouse liver samples (*n* = 4) (i.e. GSE125637) (34). Eighteen of 238 downregulated genes (7.6%) overlapped with the dataset of mouse liver samples (Fig. 6G), namely RXRG, TNNT2, FITM1, CCDC181, GPM6A, PSRC1, CKS2, TTK, BIRC5, DLGAP5, KIF2C, CDK1, CCNA2, UBE2C, HMGB2, CDC20, PLK1 and PRC1. Fifty of 431 upregulated genes (11.6%) overlapped with the dataset of mouse liver samples (Fig. 6H), namely AFF1, ZHX3, ALDH3A2, SOD2, GBF1, INSR, TXNDC11, OGFRL1, ELL2, BDH1, TSKU, GGCX, EGFR, ST6GAL1, CPEB4, HNF4A, MERTK, SLC5A9, ACOX1, TRIM14, PPARA, PPM1L, IL1RAP, CHPT1, PLXNC1, MICAL2, AR, DEPTOR, LIPC, S100A1, AK4, ABCA1, AQP3, ABCC3, KCNJ16, ACADL, SLC7A4, CLDN1, A1CF, RBP4, APOB, SERPINA7, SLC34A2, KLB, SLC10A1, ZFYVE28, C6, ABCB11, HOXC10 and HOXA9. These results suggested that our RNA-seq data were partially validated in the context of ATP7B deficiency and that the overlapping genes might be commonly regulated by ATP7B deficiency among mammalian species.

### Retinoids increased the Cp expression and secretion levels in WD-specific hepatocytes

**Figure 7:**
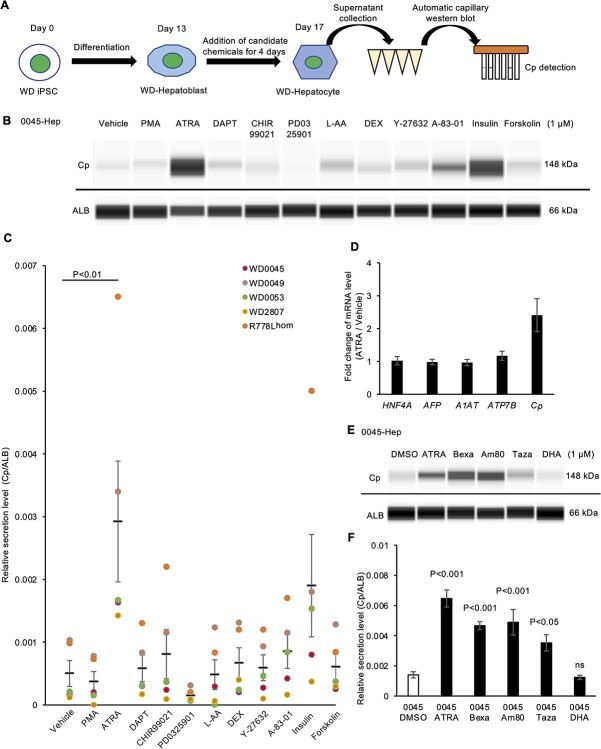
Drug screening showed that ATRA and retinoids increased the Cp expression and secretion in WD-iPSC-derived hepatocytes. (**A**) Schematic illustration of Cp secretion-based drug screening. (**B**) Representative image of western blot data concerning Cp and ALB proteins secreted by 0045-Hep on differentiation day 17 after treatment with the indicated drugs for 4 days. (**C**) Quantified data concerning the Cp secretion after normalization with ALB, dot colors indicate data from different WD-Hep. Data are shown as the mean ± SEM (*n* = 5). Statistical significance was determined by Dunnett’s test relative to DMSO-treated conditions (vehicle). (**D**) Expression of *HNF4A, AFP, A1AT, ATP7B* and *Cp* mRNA in 0045-Hep on differentiation day 17 after treatment with DMSO (vehicle) or ATRA for 4 days quantified by RT-qPCR. Data are shown as the mean ± SEM (*n* = 3). (**E**) Representative images of western blots showing the Cp secretion of 0045-Hep on day 17 after treatment with the two indicated RXR and RAR agonists for 4 days. (**F**) Quantified data of secreted Cp protein after normalization with ALB protein in 0045-Hep treated with the two indicated RXR and RAR agonists. Data are shown as the mean ± SEM (*n* = 4). *P*-values were determined by Dunnett’s test relative to the DMSO-treated condition (vehicle).

The above-mentioned findings confirmed that a decreased level of secreted Cp was a robust and convenient indicator of ATP7B dysfunction in WD-specific iPSC-derived hepatocytes. As the up-regulated Cp secretion in these WD-specific hepatocytes may aid in relieving excessive copper-induced symptoms, we developed a chemical screening system to evaluate the Cp secretion levels in iPSC-derived hepatocytes using an automatic capillary western blotting device (Fig. 7A).

A total of 11 candidate chemicals reported to affect Cp expression and/or secretion in other biological contexts were screened in these five WD-specific iPSC-derived hepatocyte sets. However, among the 11 candidate chemicals, only ATRA significantly increased the Cp secretion in these hepatocytes compared with Dimethyl sulfoxide (DMSO)-treated control conditions (Fig. 7B and C, and Supplementary Material, Fig. S6A). Although not to a significant degree, insulin also increased the Cp secretion, possibly via the regulation of hepatocyte differentiation (35). To exclude the effect of ATRA on hepatocyte differentiation, we further examined the mRNA expression of liver-related genes. RT-qPCR showed that the addition of ATRA increased the *Cp* mRNA expression without obvious changes in the expression of other hepatic genes among these five WD-specific hepatocytes (Fig. 7D). These results suggested that ATRA restored the decreased expression and secretion of Cp in WD-specific hepatocytes without affecting hepatocyte identity.

ATRA belongs to the retinoid family of molecules and regulates a wide variety of physiological functions through its nuclear receptors, known as the classical retinoic acid receptors (RARs) and nonclassical retinoid X receptors (RXRs) (36,37). To further verify and expand on the effect of ATRA on Cp secretion in WD-Hep and extrapolate the effect to clinically used retinoids, we employed four clinically approved retinoid derivatives. Tazarotene and Am80 are RAR-selective retinoids (38,39), and Bexarotene and *cis*-4,7,10,13,16,19-Docosahexaenoic acid (DHA) are RXR-selective retinoids (40,41). Effects of these four retinoids and ATRA on Cp secretion were further examined in WD-specific hepatocytes. Except for DHA, these retinoids increased Cp secretion in hepatocytes derived from iPSC lines of HPS0045, HPS0053 and R778L-homozygous mutant (Fig. 7E and F and Supplementary Material, Fig. S6B–E). These results suggested that retinoid derivatives restored the decreased Cp expression and secretion in WD-specific hepatocytes.

### ATRA suppresses oleic acid-induced ROS production in WD-specific hepatocytes

**Figure 8:**
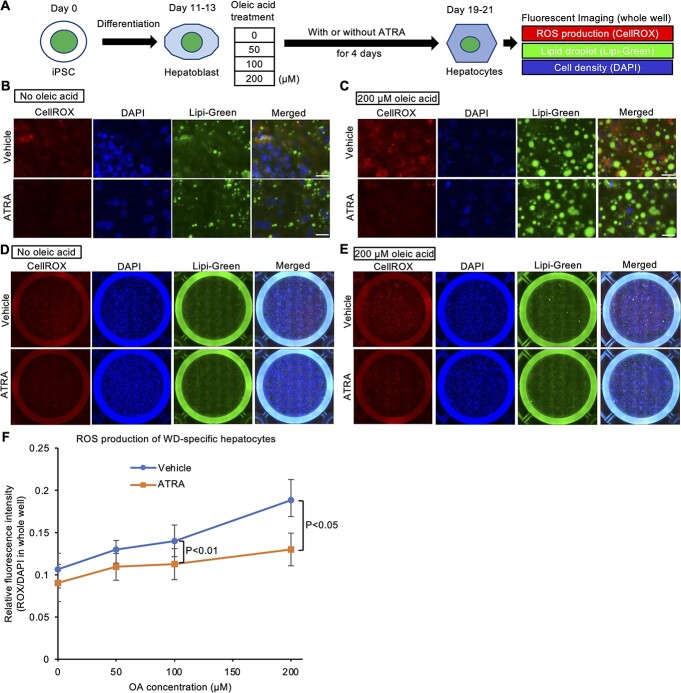
ATRA decreases oleic acid-induced ROS production in WD-iPSC-derived hepatocytes. (**A**) Schematic illustration of a cellular assay of ROS production and lipid accumulation in WD-iPSC-derived hepatocytes treated with different concentrations of oleic acid with or without ATRA. (**B** and **C**) Representative magnified lipid fluorescent images of CellROX, DAPI and Lipi-Green in HPS0045-derived hepatocytes on differentiation day 19 after oleic acid treatment at 0 μM (B) or 200 μM (C) for 8 days with or without ATRA for 4 days. Scale bar = 25 μm. (**D and E**) Representative tiled whole well fluorescent images of CellROX Deep Red, DAPI and Lipi-Green in HPS0045-derived hepatocytes after oleic acid treatment at 0 μM (E) or 200 μM (F) for 8 days with or without ATRA for 4 days. (**F**) Quantified ROS production at different concentrations of oleic acid with or without ATRA across all four WD patient-derived hepatocyte types (HPS0045, HPS0049, HPS0053 and HPS2807). Fluorescence intensity of CellROX and DAPI was calculated from tiled whole well fluorescent images. Data are shown as the mean ± SEM (*n* = 4). *P*-values were determined by two-tailed Student’s *t*-tests for paired samples from each cell line.

Our transcriptome analysis of WD-specific hepatocytes aforementioned indicated that an abnormal gene expression was related to liver dysfunction and lipid metabolism. Hepatic steatosis is one of the earliest symptoms in WD patients, being possibly caused by an abnormal lipid metabolism in response to copper accumulation in WD patients (42–44). Although the molecular mechanisms underlying the copper accumulation and abnormal lipid metabolism in WD remain elusive, ROS are known to play a central role in their mediation (43–45). Therefore, to recapitulate the features of hepatic steatosis in WD, the lipid accumulation and ROS production were evaluated in WD-specific hepatocytes treated with different concentrations of oleic acid by fluorescent imaging with specific chemical probes (Fig. 8A). In this assay system, we examined the effect of ATRA on lipid accumulation and ROS production. Lipid droplets were markedly enlarged in the WD-derived hepatocytes treated with 200 μM of oleic acid (Fig. 8B and C). The level of ROS production was also increased by the oleic acid treatment. The calculation of the fluorescent intensity from tiled whole well images across four WD patient-derived hepatocyte types (HPS0045, HPS0049, HPS0053 and HPS2807) indicated that treatment with ATRA reduced the ROS production in the oleic acid-treated WD-derived hepatocytes (Fig. 8D–F), but not in the oleic acid-treated WTC11-derived hepatocytes (Supplementary Material, Fig. S7A–C). The expression of hepatic marker genes, such as *ALB*, *AFP*, *A1AT* and *Cp*, was decreased by the addition of a high concentration (200 μM) of oleic acid in WD-specific hepatocytes but not in WTC11-derived hepatocytes (Supplementary Material, Fig. S7D and E), suggesting that the WD-specific hepatocytes being more susceptible to the effect of oleic acid than WTC11-derived ones. Our results indicated that ATRA alleviated ROS production induced by accumulated lipids in WD-derived hepatocytes.

## Discussion

In this study, we found that ATRA and several other clinically approved retinoids rescued the decreased expression and secretion of Cp in WD-specific and ATP7B-deficient hepatocytes. Furthermore, ATRA alleviated ROS production in WD-specific hepatocytes treated with elevated concentrations of oleic acid. Because Cp reduction and liver steatosis are the initial symptoms of WD, our findings suggested that retinoids were viable new therapeutic drugs for preventing the aggravation of WD. This therapeutic approach is supported by previous studies using genetically modified animals. Loss of RA signaling in mouse liver resulted in steatohepatitis and liver tumors, and feeding a high-RA diet restored the hepatic abnormalities and prevented the development of liver tumors (46). RA also exhibited suppressive effects on iron-induced oxidative stress in the same transgenic mice (47). In addition, ATRA effectively improved liver steatosis in a high-fat rabbit model (48). In Atp7b (−/−) mice, the liver X receptor (LXR)/RXR pathway activity is decreased (34), and the activation of this pathway or the feeding of zinc-enriched foods can ameliorate liver damage (16,43,49). These findings, along with those of our transcriptome analysis on WD-specific hepatocytes, which highlighted the differentially expressed genes involved in RA signaling pathways and abnormal cholesterol, fatty acid, and bile acid metabolism pathways, support the efficacy of retinoids for preventing hepatic steatosis caused by WD, as determined using patient-derived hepatocyte models.

Regarding the molecular mechanisms involved in the rescue of Cp secretion by ATRA and certain retinoid derivatives, RXR or RAR signaling pathways may mediate this process. ATRA and the other retinoid derivatives evaluated in this study bind the nuclear receptors RAR/RXR to promote down-stream gene transcription. Because the Cp mRNA expression was shown to be upregulated by ATRA treatment in WD-specific iPSC-derived hepatocytes, activated RAR/RXR may promote the transcription of the *Cp* gene directly or indirectly. The stability and export of the Cp protein are also regulated by posttranslational modifications, such as oxidation, glycosylation and a glycosylphosphatidylinositol anchor (50–52), as well as copper ion binding. The RAR/RXR signaling pathways might influence these posttranslational processes.

We generated iPSC lines from four WD patients carrying compound heterozygous mutations, along with ATP7B-defecient iPSC lines (deletion and point mutants). We also corrected the mutation in an existing iPSC line carrying the R778L homozygous mutation (17). Some of the mutations reported here had never been previously identified, nor had the complicated compound effects of these combinations of mutations been reported. Thus, their genotype–phenotype correlations were still under investigation. Previous studies using WD-specific iPSCs, which used only one patient line each, simply focused on the expression, localization and functionality of ATP7B and/or the cellular toxicity might cause to show different results (summarized in Supplementary Material, Table S4). As we have shown in this study, the expression and localization of ATP7B protein among WD-specific hepatocytes were heterogeneous. Our findings indicate that common pathological mechanisms of WD can be identified via integrative approaches using various genotypes with the *ATP7B* gene.

We found that the Cp expression and secretion were robustly decreased in WD-specific hepatocytes. Since a low plasma Cp concentration is a general clinical feature of WD, our findings represent the successful recapitulation of WD phenotypes *in vitro*. Interestingly, not only the Cp secretion but also the mRNA expression of Cp was downregulated in WD-specific hepatocytes. These results cannot be explained by the common notion that apo-Cp has weak protein stability compared with holo-Cp, where the copper ion is delivered by ATP7B. Indeed, a previous study reported that the Cp mRNA expression in liver samples from WD patients was decreased (53). Decreased Cp mRNA levels are therefore likely attributed to other mechanisms in WD-specific hepatocytes. In addition, the Cp expression was reported to be decreased by an mRNA decay mechanism in response to intracellular oxidative stress (54). Thus, the decreased Cp mRNA expression in WD-specific hepatocytes might have been caused by abnormal oxidative stress and/or nuclear receptor signaling pathways.

We developed a Cp secretion-based drug screening assay and a ROS detection assay in response to high oleic acid concentrations using human iPSC-derived hepatocytes. Our approach may be a faithful recapitulation of the initial cellular features of WD-specific hepatocytes and thus provide an effective platform for developing therapeutic technologies for hepatic steatosis in WD and other fatty liver diseases, as well as for examining the molecular pathogenesis underlying WD. Because the detection methods are clear and easy to apply, these assays should be further adapted to allow high-throughput screening systems to identify ideal therapeutic drugs in the future.

Although we demonstrated that retinoids rescued Cp secretion and alleviate oxidative stress in WD-specific hepatocytes for the first time, animal tests and preclinical evaluations will be required before clinical trials can be performed. In addition, although we used iPSC lines derived from sufficient numbers of WD patients to perform statistical tests to draw general conclusions, there might have been outliers due to natural variations among WD patients with different types of mutations. Finally, the molecular mechanism underlying how ATP7B deficiency leads to an abnormal lipid mechanism remains unclear. Future studies should investigate the relationships between copper and lipid metabolism.

## Materials and Methods

### Human subjects

The generation and use of human iPSCs were approved by the Ethics Committees of RIKEN BioResource Research Center, Tokyo Metropolitan Institute of Medical Science, and Graduate School of Medicine of Kyoto University. Formal informed consent was obtained from the patients.

### Animal experimentation

All animal experiments were approved by the Animal Experimentation Committee at the RIKEN Tsukuba Institute and performed according to the committee’s guiding principles and the ‘Guide for the Care and Use of Laboratory Animals’ published by the National Institutes of Health.

### iPSC generation and culture

In this study, WTC11 (GM25256 from Coriell Institute) (55), 606A1 (HPS0328 from RIKEN Cell Bank) (56), 454E2 (HPS0077 from RIKEN Cell Bank), 409B2 (HPS0076 from RIKEN Cell Bank) and 19–9-7 T (iPS DF19–9-7 T from WiCell) (57) were used as HD iPSC lines. As WD patient-specific iPSC lines, HPS0045 (HiPS-RIKEN-5A from RIKEN Cell Bank), HPS0049 (HiPS-RIKEN-7A from RIKEN Cell Bank), HPS0053 (HiPS-RIKEN-9A from RIKEN Cell Bank), HPS2807 (RIKEN Cell Bank) and R778L homozygous donor (a gift from Drs. Esteban and Pei) (17) were used. In this study, the HPS0045, HPS0049 and HPS0053 iPSC lines were generated from skin fibroblasts deposited in RIKEN Cell Bank as RCB0395 NCU-F8, RCB0390 NCU-F3 and RCB0391 NCU-F4, respectively, obtained from one WD patient each. These iPSC lines were reprogrammed with retroviral vectors carrying OCT3/4, SOX2, KLF4 and MYC, according to a previously reported protocol (58). The HPS2807 WD iPSC line was generated from peripheral blood mononuclear cells obtained from a WD patient at Kyoto University. This line was reprogrammed with episomal vectors carrying OCT3/4, SOX2, KLF4, MYCL1, Lin28, mp53DD and EBNA1, according to the previous protocol (56). Episomal vectors integration was inspired with EBNA-1 DNA copy number detection in the genome DNA of each iPSC line. A feeder-free culture system was used to culture all of the iPSC lines (59). The iPSCs were cultured on 0.25 μg/cm^2^ iMatrix-511 silk (Matrixome, Osaka, Japan) with StemFit AK02N medium (Ajinomoto). Cells were fed every other day and passaged at 80–90% confluency after 6–8 days of culture. EDTA solution (0.5 mM) was used to dissociate sheets into individual cells, and the resulting solution was then seeded at a density of 2500/cm^2^. Y-27632 (10 μM; Wako) was used on the cell seeding day. All of the iPSC lines used in this study were confirmed to be mycoplasma-negative. Short tandem repeat-PCR was performed for all iPSC lines to match the donor samples.

Information on donors and methods of generating iPSCs is shown in Supplementary Material, Table S1.

### Microarray assays

Copy number variations in WD iPSC lines were detected with a microarray assay using the CytoScan Optima Suite (Thermo Fisher Scientific). A CytoScan Optima assay was performed according to the manufacturer’s protocol. Before the assay, gDNA was extracted from WD-specific iPSCs with a DNeasy Blood & Tissue kit (Qiagen). The resulting CytoScan Optima arrays were read with a GeneChip System 3000. Data were analyzed with the Chromosome Analysis Suite and Affymetrix GeneChip Command Console software programs.

### Pluripotency identification with teratoma formation

WD iPSC lines were dissociated with 0.5 mM ethylenediaminetetraacetic acid (EDTA) in Dulbecco’s phosphate-buffered saline (DPBS) and then resuspended with 1 ml Stem Fit AK02N complete medium (Ajinomoto) supplemented with 10 μM Y-27632 (Wako). The cell suspension medium containing 1 × 10^6^ cells was transferred to a 1.5-ml tube. Cells were collected by centrifuging at 200*g* for 3 min and then resuspended in Stem Fit AK02N complete medium (Ajinomoto) supplemented with 10 μM Y-27632 containing 50% Matrigel solution. The cell suspension was aspirated in a 1-ml syringe with an 18-G needle and kept on ice until injection. Cells were injected into the legs and testis of NOD.CB17-Prkdc^scid^/J, 4-week-old male mice, 1 × 10^6^ cells per injection (Charles River Laboratories). Six to 10 weeks later, teratomas were collected from the legs or testis, as appropriate, and fixed with 4% paraformaldehyde dissolved in DPBS (Nacalai Tesque) for 1 week at 4°C, followed by further storage in 70% ETOH at 4°C until sectioning. Paraffin-embedded sections were made and processed for hematoxylin and eosin (HE) staining by Genostaff, Inc. Three germ layer derivatives were observed using a BZ-X800 microscope (KEYENCE).

### Pluripotency identification with EB formation

WD iPSC lines were prepared as above. A total of 1.0 × 10^4^ cells were seeded in a 96-well-V plate (EZ-BindShut) in each well with 100 μl Stem Fit AK02N complete medium (Ajinomoto) supplemented with 10 μM Y-27632 (Wako). Cells were centrifuged at 200*g* for 3 min before culture. The next day, the culture medium was switched to DMEM high Glucose (Gibco) supplemented with 10% fetal bovine serum (FBS) (EB medium). Seven days later, EBs were transferred into 0.1% (w/v) Gelatin Solution (Wako)-coated 24-well plates and further cultured in EB medium for another 7 days. Cells were fed every other day. Pluripotency was validated by evaluating the expression of the three germ layer markers (TUJ1/SMA/AFP) with immunostaining.

### Mutation identification in WD-Hep

To identify the ATP7B mutations in each WD iPSC line, WD patient iPSC-derived hepatocytes were collected around day 17, and total RNA was extracted with a FastGene RNA premium kit (Nippon Genetics). cDNA was synthesized by reverse transcription (RT). Less than 1 μg of mRNA was further used for first-strand cDNA synthesis using a ReverTra Ace qPCR RT kit (Toyobo). To synthesize whole length transcripts of the *ATP7B* gene, oligo dT was used in the RT reaction. The thermal conditions for the RT reaction were as follows: 30°C for 10 min, 42°C for 60 min and 99°C for 5 min. Ten pairs of primers were designed on the basis of transcript variant 1 (GenBank Accession Number: NM_000053.4) and used to amplify the whole coding sequence of ATP7B with Tks Gflex DNA Polymerase (Takara). Primer sequences are listed in Supplementary Material, Table S5. The thermal cycling conditions were as follows: initial denaturation at 94°C for 1 min, 35 cycles of 3-step thermo-cycling (denaturation at 98°C for 10 s, annealing at 60°C for 15 s and extension at 68°C 60 s) and then hold at 4°C. PCR products were confirmed by agarose gel electrophoresis before a sequence analysis. PCR products used for sequencing were prepared with a FastGene Gel/PCR extraction kit (Nippon Genetics).

Mutations in each WD iPSC line were further verified in the respective gDNA sample. gDNA was prepared using a DNeasyBlood & Tissue Kit (Qiagen). PCR primer sequences used for the *ATP7B* genome sequence are listed in Supplementary Material, Table S5. Thermal cycling conditions were the same as above. Sanger sequence analyses were further performed with a Genetic Analyzer 3130 (Applied Biosystems) to identify the mutations in each fragment. All of the experiments were performed following the manufacturer’s instructions for each respective kit.

### Genome editing

The ATP7B exon1-deletion iPSC line was generated by Cas9 targeting of the start codon to introduce a deletion mutation. The gRNA sequence used was 5′-GTGCGGGACGATGCCTGAGC-3′. WTC11 cells were treated with Accutase (Innovative Cell Technologies) and resuspended in PBS to count the cell number. An aliquot of the cell suspension containing 2 × 10^5^ cells was transferred into a 1.5-ml tube, and the cells were spun down. We resuspended the cells in 20 μl of P3 Primary Cell Nucleofector Solution (Lonza) containing 500 ng of pX330 (#42230; Addgene) with gRNA. The program DS-138 was executed by a 4D-Nucleofector System (Lonza), and then the cells were cultured with 10 μM Y-27632. Clones were isolated by repeated serial dilutions while detecting the introduced mutation via droplet digital PCR (ddPCR), as described previously (60,61).

ATP7B R778L point mutagenesis was conducted first by a pair of TALENs (ATP7B-TALEN F and R) to generate a heterozygous line, as described previously (61). The target sequences of ATP7B-TALEN F and R were 5′-TGTGTTCATTGCCCTGGG-3′ and 5′-TGCTGTTACCTTTGCCAA-3′, respectively. The single-stranded donor DNA was 5′-CATGCTCTTTGTGTTCATTGCCCTGGGCCTGTGGCTGGAACACTTGGCAAAGGTAACAGC-3′ (underlined letter indicates the mutation site). We introduced 125 ng of each TALEN and 250 ng of the single-stranded donor DNA to isolate a heterozygously mutated clone. The isolated heterozygous clone was retargeted with the WT-specific gRNA and Cas9 to convert the WT allele into the R778L allele with a SM [codon substitution from CGU (Arg) to CUC (Leu)]. The gRNA sequence and the single-stranded oligonucleotide donor DNA were 5′-gTTGTGTTCATTGCCCTGGGC-3′ and 5′-CATGCTCTTTGTGTTCATTGCCCTGGGCCTCTGGCTGGAACACTTGGCAAAGGTAACAGC-3′, respectively (lower case g indicates a guanine introduced to maximize the gRNA expression via the U6 promoter, and underlined letters indicate the mutation site). We introduced 250 ng of pX330 with this gRNA and 250 ng of the oligonucleotide donor DNA to isolate a homozygous clone. The procedures for nucleofection and clone isolation were the same as those used for the ATP7B-exon1-deletion iPSC line.

The ATP7B R778Lhet iPSC line was generated using Cas9-D10A nickase targeting the mutation site and double-stranded oligonucleotide donor DNA to correct the mutation. The gRNA and donor sequences used were 5′-gCATTGCCCTGGGCCTGTGGC-3′ and 5′-CATGCTCTTTGTGTTCATTGCCCTGGGCCGGTGGCTGGAACACTTGGCAAAGGTAACAGC-3′, respectively (lower case g indicates a guanine introduced to maximize the gRNA expression via the U6 promoter, and underlined letters indicate the mutation site). We introduced 250 ng of pX335 (#42335; Addgene) with this gRNA and 250 ng of the donor DNA into the ATP7B R778Lhom iPSC line to correct the mutation. The procedures for nucleofection and clone isolation were the same as aforementioned. The ddPCR probes and primers to detect the genome editing events are listed in Supplementary Material, Table S6.

### TA cloning

To clarify the allelic location of these heterozygous mutations in each WD iPSC line, the Mighty Cloning Reagent Set (Blunt End; Takara) was used to clone the targeted PCR products of the *ATP7B* gene into pUC118Hinc ll/BAP, a blunt-end cloning vector containing a multiple cloning site, followed by credible blue/white colony selection. The PCR products were ligated into pUC118 plasmid, which were further transformed into DH5alpha competent cells. After 1 h shaking culture at 280 rpm in SOC medium at 37°C, the transformed *Escherichia coli* was spread on LB agar (Nacalai Tesque) plate and incubated at 37°C overnight. Single colonies were selected and incubated in 3 ml of LB medium (Nacalai Tesque) at 280 rpm for 8 h. Plasmids were prepared with a FastGene miniprep plasmid extraction kit (Nippon Genetics). The plasmid concentration was determined by Nanodrop. All of the experiments were performed following the manufacturer’s instructions for each kit. The cloned inserts were sequenced through PCR using M13 primer FW: GTAAAACGACGGCCAGT and M13 primer RV: CAGGA AACAG CTATG AC by Sanger sequencing at Fasmac or Eurofin Genomics. Similarly, for genotyping of ATP7B-exon1-deletion iPSC, the gDNA sequence around ATP7B exon 1 was amplified by PCR using ATP7B Def seq FW: CGCAACTTTGAATCATCCGTGTGA and ATP7B Def seq RV: GAGATAAAGTGAGCGTCGAGTTGC for TA-cloning. The cloned inserts in plasmids were sequenced by Sanger sequencing at Eurofins Genomics using M13 M4 primer: GTTTTCCCAGTCACGAC and M13 primer RV.

### qRT-PCR

Total RNA preparation and first-strand cDNA synthesis protocol were the same as above. Random primer was used for first-strand cDNA synthesis. Real-Time qPCR reactions were performed with a QuantStudio 3 System (Applied Biosystems) using THUNDERBIRD Probe qPCR Mix (Toyobo) with TaqMan probes according to the manufacturer’s instructions. The gene expression was described as the fold change relative to the control sample value (ΔΔCt method) after being normalized to corresponding GAPDH values. The TaqMan probes used for the RT-qPCR experiments are listed in Supplementary Material, Table S7.

### Immunocytochemistry

Immunocytochemistry was performed to check the expression of pluripotency markers (OCT4/NANOG/SOX2/KLF4/SSEA-4), endoderm-related markers (SOX17/GATA6) and hepatocyte-related markers (HNF4A/AFP/ALB). In addition, immunocytochemistry of ATP7B and p230 trans-Golgi in WD-iPSC-derived hepatocytes was performed using iPSC-derived hepatocytes on differentiation day 13 or 15. In brief, cells were fixed with PBS containing 4% paraformaldehyde for 10 min at room temperature. To stain transcription factors, cells were permeabilized in PBS containing 0.1% Triton X-100 for another 10 min at room temperature and then washed with PBS. Primary antibodies were incubated in 0.1% FBS in PBS overnight at 4°C. The primary antibodies used in this study are listed in Supplementary Material, Table S8. The secondary antibodies were incubated for 1 h at room temperature in 0.1% bovine serum albumin (BSA) in PBS. The secondary antibodies used in this study are listed in Supplementary Material, Table S9. Cell nuclei were stained with Fluoro-KEEPER Antifade Reagent DAPI (Nacalai Tesque). Images were taken with an all-in-one fluorescent microscope (BZ-X800; KEYENCE).

### Hepatocyte differentiation

A stepwise differentiation protocol was adopted to differentiate iPSCs into hepatocytes in this study, as modified from a previous study (31). For differentiation preparation, iPSCs were dissociated with 0.5 mM EDTA in PBS, and then 2.5 × 10^4^ iPSCs were seeded in each well of a 24-well plate with StemFit complete culture medium (Ajinomoto) supplemented with 10 μM Y-27632 (Wako) and 0.25 μg/cm^2^ iMatrix-511 silk (Matrixome) on day 0. Definitive endoderm induction was performed from day 1 to day 2. The culture medium was changed to StemFit (without supplement C) supplemented with 3 μM CHIR99021 (Wako) and 10 ng/ml Activin A (Wako) on day 1. The culture medium was switched to StemFit (without supplement C) on day 2.

Hepatoblast induction was performed from days 3 to 6. During this stage, cells were cultured in StemSure DMEM (Wako) supplemented with 20% Stemsure serum replacement (SSR; Wako), 1% L-alanyl-L-Glutamine (GlutaMax) solution (Naclai Tesque), 1% 50 mM Monothioglycerol (MTG) Solution (Wako), 1% MEM nonessential amino acids (NEAAs; Nacalai Tesque), 1% DMSO solution (Sigma Aldrich), 5 μM DAPT (Calbiochem), 0.3 μM A83–01 (SIGMA) and 1% Penicillin (10000 U/ml)/Streptomycin (10000 μg/ml) solution (Nacalai Tesque). The culture medium was changed every other day.

Hepatocyte induction was performed from days 7 to 14. During this stage, cells were cultured in DMEM/Ham’s F12 (Nacalai Tesque) supplemented with 1% SSR (Wako), 1% Glutamax solution (Naclai Tesque), 2% B27 minus insulin (Gibco), 1% NEAA (Nacalai Tesque), 10 ng/ml recombinant human Hepatocyte Growth Factor (rhHGF; Wako), 10 ng/ml recombinant human Oncostatin M (rhOSM; Wako), 5 μM DAPT (Calbiochem), 0.3 μM A83–01 (SIGMA), 10 μM Dexamethasone (Nacalai Tesque) and 1% Penicillin/Streptomycin solution (Nacalai Tesque). The culture medium was changed every other day.

Hepatocyte maturation was performed from day 15. During this stage, cells were washed with D-PBS twice, and the culture medium was switched to Hepatocyte Basal Medium (HBM) (Lonza) supplemented with 20 ng/ml rhHGF (Wako), 20 ng/ml rhOSM (Wako), 10 μM Dexamethasone (Nacalai Tesque), 1% P/S (Nacalai Tesque), 5 μM DAPT (Calbiochem) and 0.3 μM A83–01 (SIGMA).

### Cell lysate and culture supernatant preparation for simple Wes

To detect ATP7B protein, iPSC-derived hepatocytes were collected on day 17. The cell lysate was prepared with sodium dodecyl sulfate-polyacrylamide gel electrophoresis Sample Buffer Solution without 2-ME (2x) (Nacalai Tesque) supplemented with 10% 1 M dithiothreitol (DTT) solution (Nacalai Tesque) and 1% protease inhibitor cocktail solution (Nacalai Tesque). To detect secreted Cp, cell culture supernatant was collected from iPSC-derived hepatocytes on day 17 and then centrifuged at 6000 rpm for 3 min. Protein concentrations were determined with Pierce 660 nm Protein Assay Reagent (Thermo Scientific) and a rainbow sunrise microplate reader (TECAN).

### Automatic capillary western blot

Western blotting was performed with a capillary automatic western blot device (Simple Western, Wes; ProteinSimple). For the detection of total ALB, ATP7B, and Cp, a Jess/Wes 12- to 230-kDa separation module for Wes, 8X25 capillary cartridges (ProteinSimple) and Anti-Rabbit/Goat/Mouse Detection Module kit (ProteinSimple) were used. Sample loading and reagent preparation were all conducted according to the manufacturer’s instructions. For the supernatant, around 10 μg of the total protein amount was loaded into each well. For the cell lysate, around 3 μg of the total protein amount was loaded into each well. For preloading preparation, samples were diluted with Simple Western 0.1× sample dilution buffer and then mixed with 1/5 volume Fluorescent Standards Mix containing DTT and denatured at 95°C for 5 min. The following primary antibodies were used to blot the target protein: anti-GAPDH, anti-ATP7B, anti-human ALB, and anti-Cp. Detailed information on the primary antibodies is listed in Supplementary Material, Table S8.

Chemiluminescent signal was detected and quantitated automatically by a Simple Western system. The compass for simple Western software program (ProteinSimple) was used to process and analyze the data. The area of the blot bands was quantified as the protein expression. To normalize the ATP7B expression in the same sample, a housekeeping protein (GAPDH) was used as the reference. To normalize the Cp secretion in different samples, secreted ALB protein was used as the reference.

### Cp secretion-based drug screening

Several different chemicals were used as candidates to increase the Cp secretion in WD-specific iPSC-derived hepatocytes, as follows: PMA, ATRA, DAPT, CHIR99021, PD0325901, L(+)-Ascorbic Acid, Dexamethasone, Y-27632, A8301, insulin and forskolin. All of these drugs were administered to the cells at a concentration of 1 μM. Drug treatment was started from differentiation day 13. To eliminate the effect of serum and undefined factors, hepatocyte cultures were washed with D-PBS twice before being switched to drug screening culture medium on day 13, which comprised HBM (Lonza) supplemented with rhHGF (Wako) 20 ng/ml and rhOSM (Wako) 20 ng/ml containing the respective drugs. Cells were fed once with the same medium and drugs on day 15. On differentiation day 17, cell culture supernatants were collected to evaluate the secreted Cp and ALB under each culture condition with the automatic capillary western blot device aforementioned.

Regarding the two RXR-selective agonists (Bexarotene and *cis*-4,7,10,13,16,19-DHA) and RAR-selective agonists (Tazarotene and Am80), the treatment and sample collecting protocol were the same as aforementioned. Information concerning the chemicals used in this study is listed in Supplementary Material, Table S10.

## RNA-Seq

Total RNA of 12 samples was extracted with a FastGene RNA premium kit (Nippon Genetics). Strand-specific library preparation was performed. The prepared library was sequenced by a NovasSeq6000 (Illumina). Sequencing was performed in a 2 × 150-bp PE configuration with a data output of about 6 Gb per sample (equivalent to about 20 million paired reads). The quality scores (Q30) of all of the samples were >94%. Library preparation and sequencing were performed in GENEWIZ. The sequencing data were analyzed with a CLC genomics workbench (QIAGEN) to identify differentially regulated genes and draw a volcano plot. The extracted genes were analyzed for enrichment in gene ontology and biological pathways using the Enrichr web program (https://maayanlab.cloud/Enrichr/). In addition, GSEA was performed using the GSEA v4.2.2 software. All gene set files for this analysis were obtained from GSEA website (www.broadinstitute.org/gsea/). Enrichment map was used for visualization of the GSEA results with enrichment score (ES) and FDR value. To perform an integrative analysis with a dataset from a microarray analysis on mouse liver samples of 3-month-old C57Bl/6 and 3-month-old Atp7b^−/−^ C57Bl/6 mice (GSE125637), differentially regulated genes were extracted from four samples each with the GEO2R web program (https://www.ncbi.nlm.nih.gov/geo/geo2r/?acc=GSE125637). To identify and count the overlapping differentially regulated genes in WD-specific hepatocytes and ATP7B KO mouse liver samples, COUNTIF functions were used with the Microsoft Excel software program (Microsoft).

### Oleic acid exposure and retinoids, RXR/RARs agonists treatment

Oleic acid exposure was started from differentiation day 11 or 13 and conducted for 8 days. Cells were cultured in third-stage differentiation medium containing oleic acid (SIGMA) at 0, 50, 100 or 200 μM. To test the inhibitory function of ATRA and RXR/RARs agonists on ROS production, ATRA and the indicated agonists were added for the last 4 days before sampling. CellROX Deep Red Reagent (Thermo Scientific) and Lipi-Green reagent (Dojindo) were used to detect the production of ROS and lipid droplets in WD-Hep, respectively. The production of ROS and lipid droplets in hepatocytes was visualized with an all-in-one fluorescent microscope (BZ-X800; KEYENCE). Cell nuclei were stained with Fluoro-KEEPER Antifade Reagent DAPI (Nacalai Tesque). Tiled whole well fluorescent images were taken for ROS production as well as lipid accumulation evaluation. The integrated density (Intden) of the entire well fluorescence was measured using the Image J software program, 1.53.e after background subtraction. To normalize the quantity of ROS and lipid droplets in different wells, the Intden of DAPI in the entire well was used as the reference.

### Statistical analyses

In this manuscript, data are shown as the mean ± the standard error of the mean (SEM) in all graphs generated using the Microsoft Excel software program for Mac (version 16.48; Microsoft). For the comparison of two samples, *P*-values were determined by an unpaired two-tailed Student’s *t*-test using the Microsoft Excel software program for Mac (version 16.48; Microsoft). For the comparison of more than two samples, *P*-values were determined by Dunnett’s test using the R software program (version 4.0.5).

## Supplementary Material

SupplementaryInformation-SD-YH_proof_ddac080

TableS2_ddac080

TableS3_ddac080

## Data Availability

RNA-seq data were deposited in NBDC human database (hum0302.v1). All of the reagents used in this paper are listed in Supplementary Material, Table S11.
