# Supplementary material for: Retinoids rescue ceruloplasmin secretion and alleviate oxidative stress in Wilson’s disease-specific hepatocytes
Source: Hum Mol Genet. 2022 Apr 7;31(21):3652–71. doi: 10.1093/hmg/ddac080 (PMC9616579; doi:10.1093/hmg/ddac080)
Supplement: SupplementaryInformation-SD-YH_proof_ddac080 [file supplementaryinformation-sd-yh_proof_ddac080.pdf]

Supplementary Materials

Retinoids rescue ceruloplasmin secretion and alleviate oxidative stress in Wilson's disease-specific hepatocytes

Dan Song, Gou Takahashi, Yun-Wen Zheng, Mami Matsuo-Takasaki, Jingyue Li, Miho Takami, Yuri An, Yasuko Hemmi, Natsumi Miharada, Tsuyoshi Fujioka, Michiya Noguchi, Takashi Nakajima, Megumu K. Saito, Yukio Nakamura, Tatsuya Oda, Yuichiro Miyaoka, and Yohei Hayashi

Corresponding authors:

Yohei Hayashi (yohei.hayashi@riken.jp) or Yuichiro Miyaoka (miyaoka-yi@igakuken.or.jp)

Table of content:

Supplementary Material, Figure S1-8

Supplementary Material, Table S1, S4-11

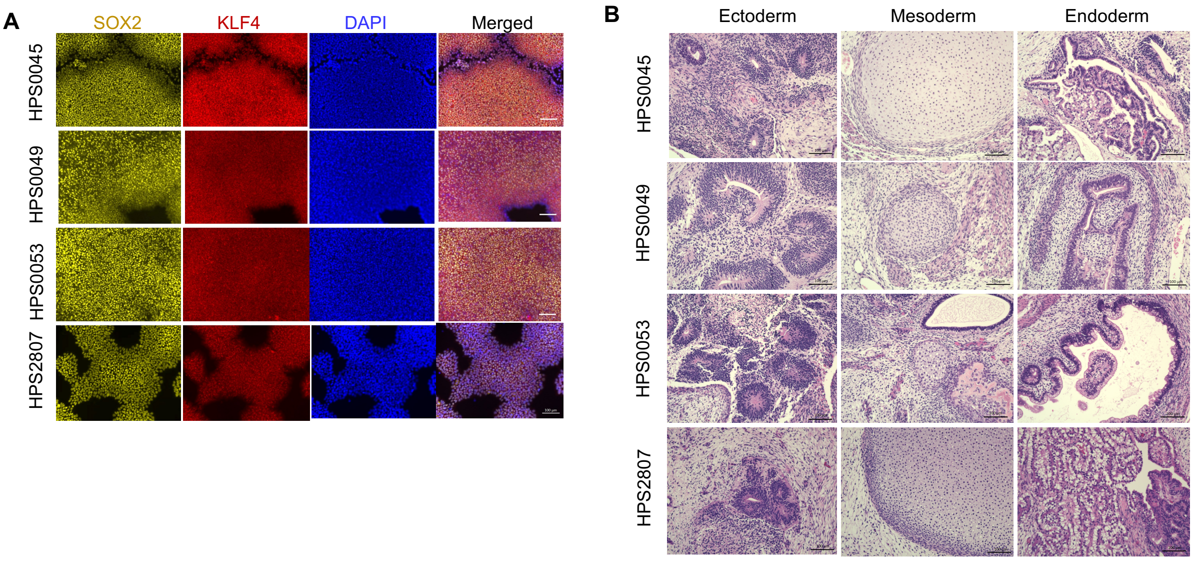

Supplementary Material, Figure S1. Characterization of WD-iPSCs established in this study, related to Fig 1. (A) Expression of self-renewal markers, SOX2 (yellow) and KLF4 (red), in WD-iPSCs. Scale bar=100 µm. **(B)** Pluripotency was evaluated with a teratoma formation assay. Three germ layers derivatives are shown. Scale bar=100 µm.

A

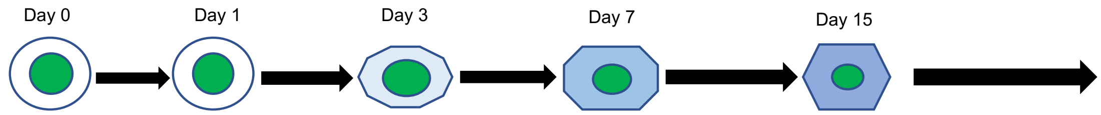

Medium name	Maintenance medium	Definitive endoderm induction medium	Hepatoblast induction medium	Hepatocyte induction medium	Hepatocyte maturation medium
Basal medium	StemFit AK02N	StemFit AK02N	Stemsure DMEM	DMEM/F12	HBM
Additives	Supplement B, Supplement C, 10 μM Y-27632 0.25 ug/cm^2 iMatrix-511 silk	Supplement B <u>Only on day 1</u> 3 μM CHIR99021, 10 ng/ml Activin A	20% SSR, 1% NEAA, 1% Glutamax, 1% MTG, 1% DMSO, 5 μM DAPT, 0.3 μM A83-01	1% SSR, 2% B27 minus insulin, 20 ng/ml HGF, 20 ng/ml OSM, 10 μM Dexamethasone, 5 μM DAPT, 0.3 μM A83-01	20 ng/ml HGF 20 ng/ml Oncostatin M 10 μM Dexamethasone 5 μM DAPT, 0.3 μM A83-01

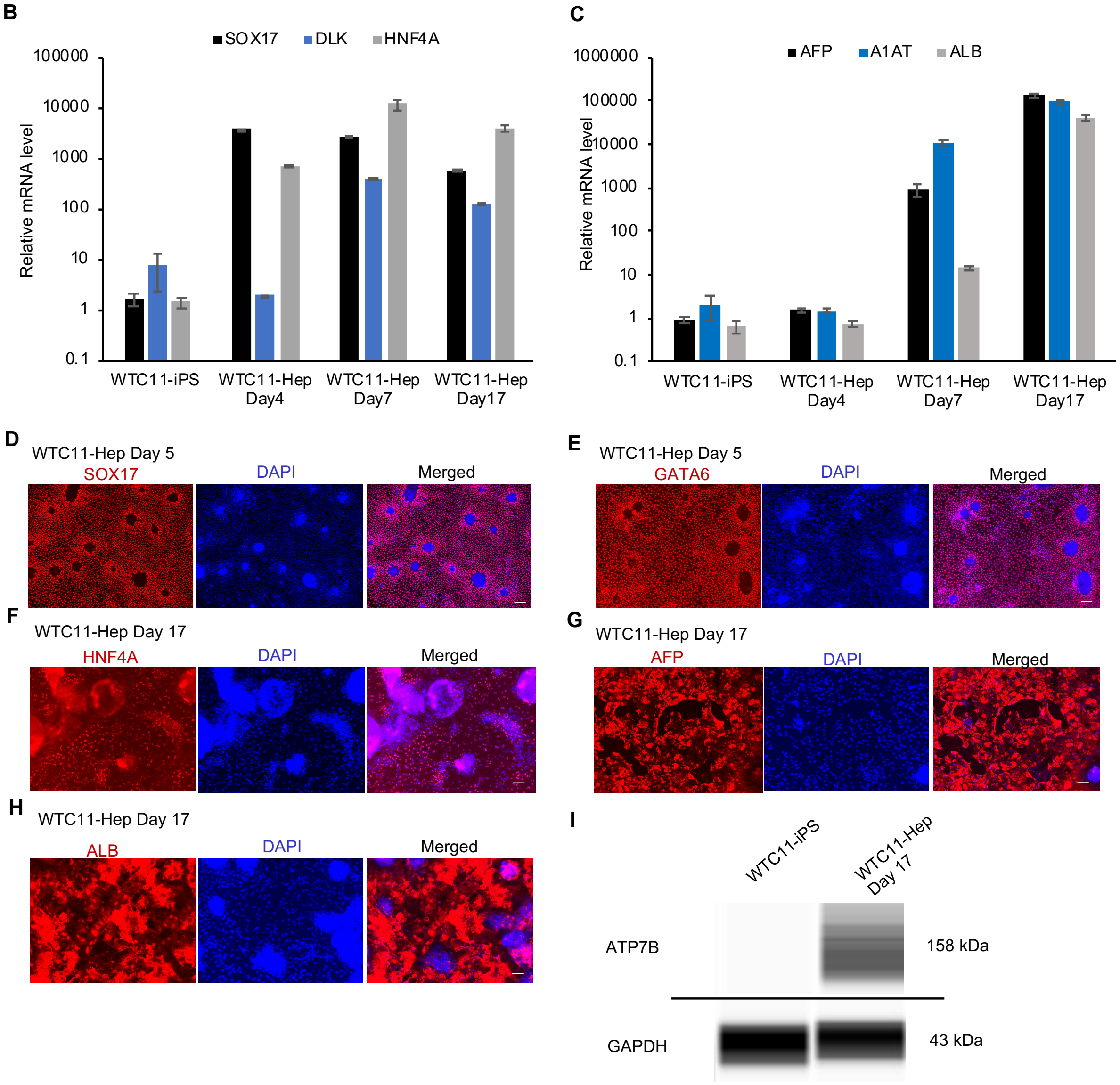

Supplementary Material, Figure S2. Methods of direct differentiation into hepatocytes used in this study, related to Fig. 2. (A) Schematic illustration showing the differentiation of hepatocytes from human iPSCs. **(B and C)** Expression of differentiation marker genes of definitive endoderm, hepatoblasts, and hepatocytes on differentiation days 0, 4, 7, and 17 detected by RT-qPCR. Bar graphs show the mean ± SEM (n=3). **(D and E)** Representative images of immunostaining showing SOX17 (D) and GATA6 (E) protein expression on

differentiation day 5 of WTC11-derived hepatocyte culture. **(F-H)** Representative images of immunostaining showing HNF4A (F), AFP (G), and ALB (H) protein expression on differentiation day 17 of WTC11-derived hepatocyte culture. Scale bar=100 μm. (I) Band images of Western blotting against ATP7B and GAPDH proteins in WTC11-iPSCs and WTC11-Hep on differentiation day 17.

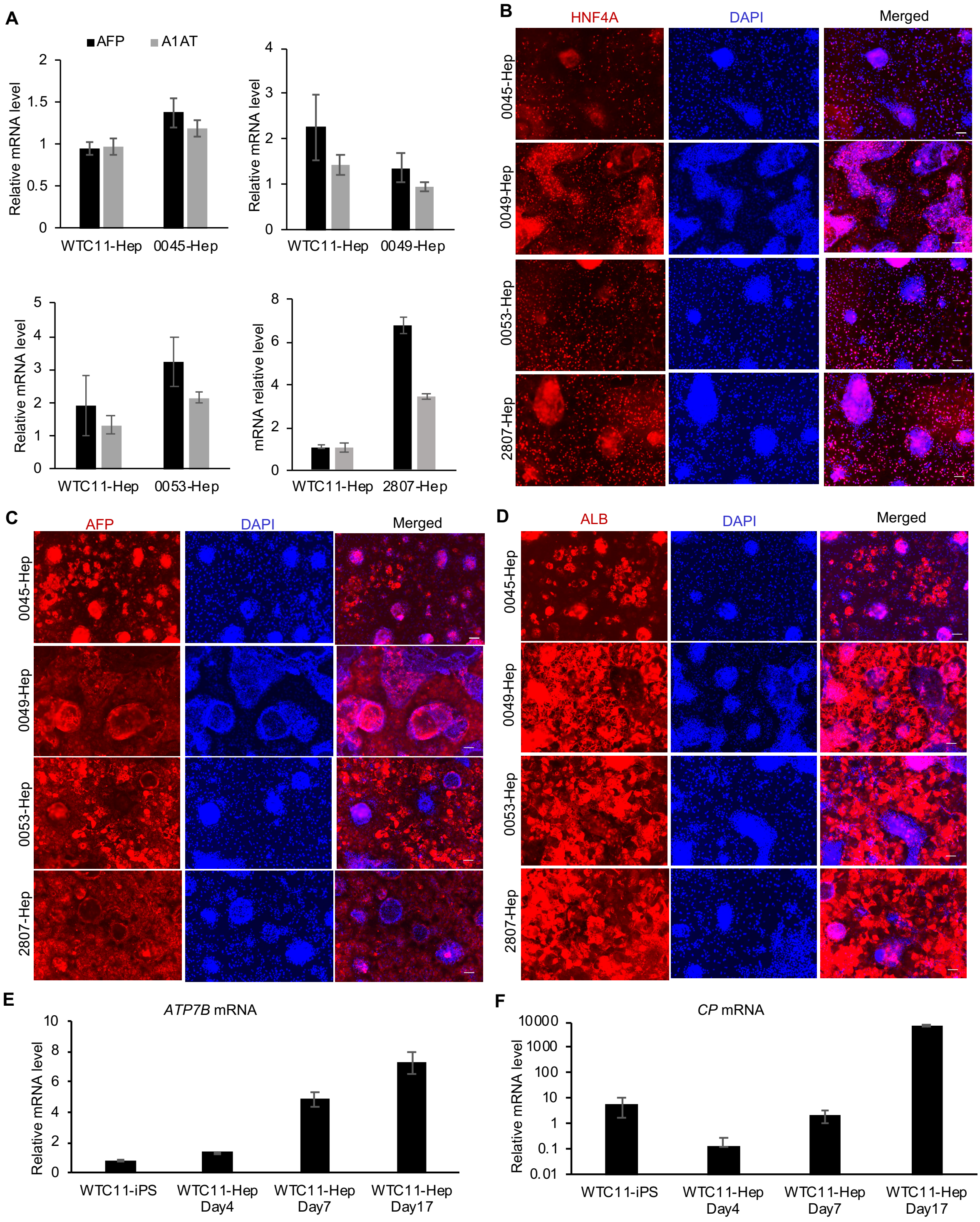

Supplementary Material, Figure S3. Hepatic differentiation capability and efficiency of WD-iPSCs generated in this study, related to Fig. 2. (A) Expression of differentiation marker genes of hepatocytes on differentiation day 17 detected by RT-qPCR. Bar graphs show the mean ± SEM (n=3). **(B-D)** Representative images of immunostaining showing HNF4A (B), AFP (C), and ALB (D) protein expression on differentiation day 17 of WTC11-derived hepatocyte culture. Scale bar=100 μm. **(E and F)** The expression of *ATP7B* (E) and *CP* (F)

mRNA during hepatic differentiation was detected by RT-qPCR (n=3). Bar graphs show the mean ± SEM.

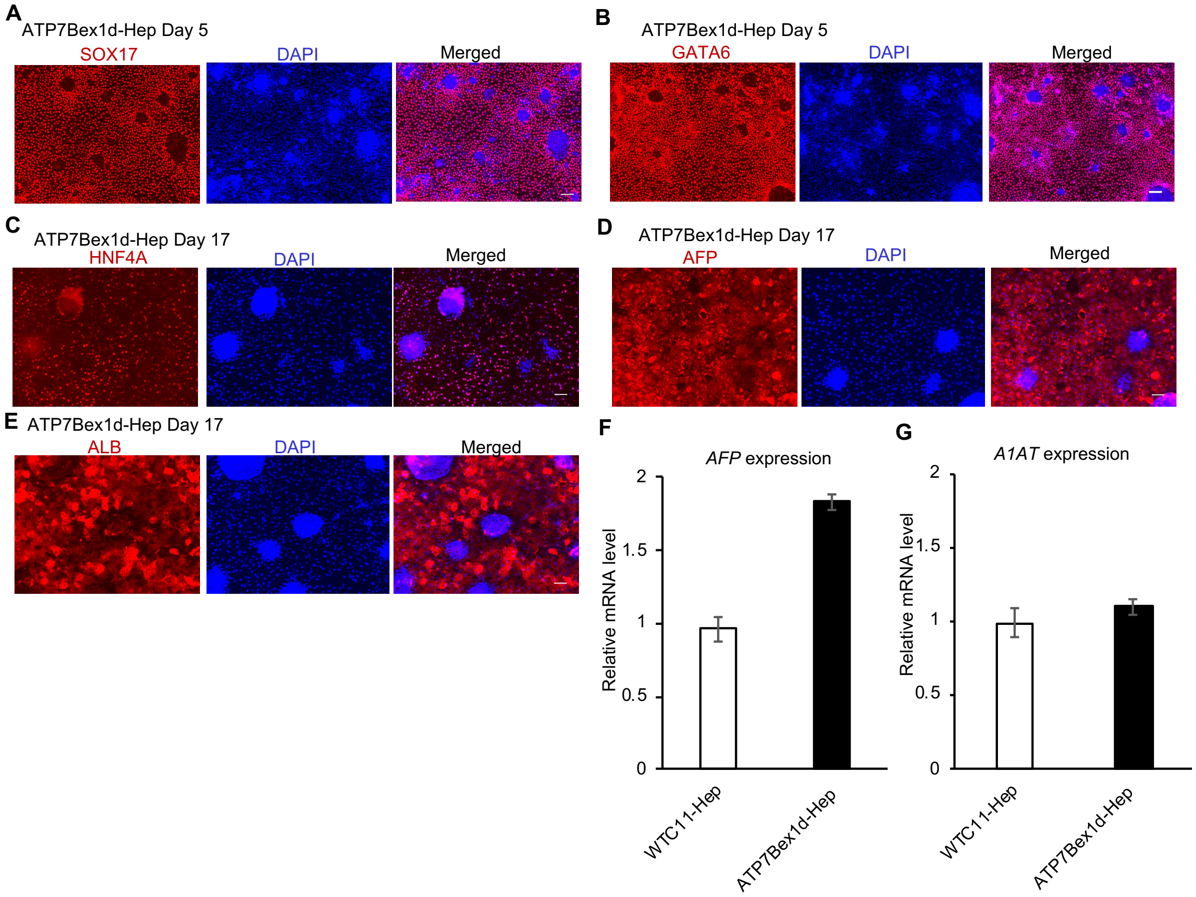

Supplementary Material, Figure S4. Hepatocyte differentiation ability of WTC11-derived *ATP7B*ex1d iPSCs, related to Fig. 3. (A and B) Representative immunostaining images showing the expression of endoderm markers SOX17 (A) and GATA6 (B) on differentiation day 5 of ATP7Bex1d-iPSCs. Scale bar=100 µm. **(C-E)** Representative immunostaining images showing the expression of hepatocyte markers HNF4A (C), AFP (D), and ALB (E) on differentiation day 17 of ATP7Bex1d-iPSCs. Scale bar=100 µm. **(F and G)** The gene expression of hepatic markers *AFP* (F) and *A1AT* (G) detected by RT-qPCR in hepatocytes derived from parental WTC11 and ATP7Bex1d iPSC lines (n=3). Bar graphs show the mean ± SEM.

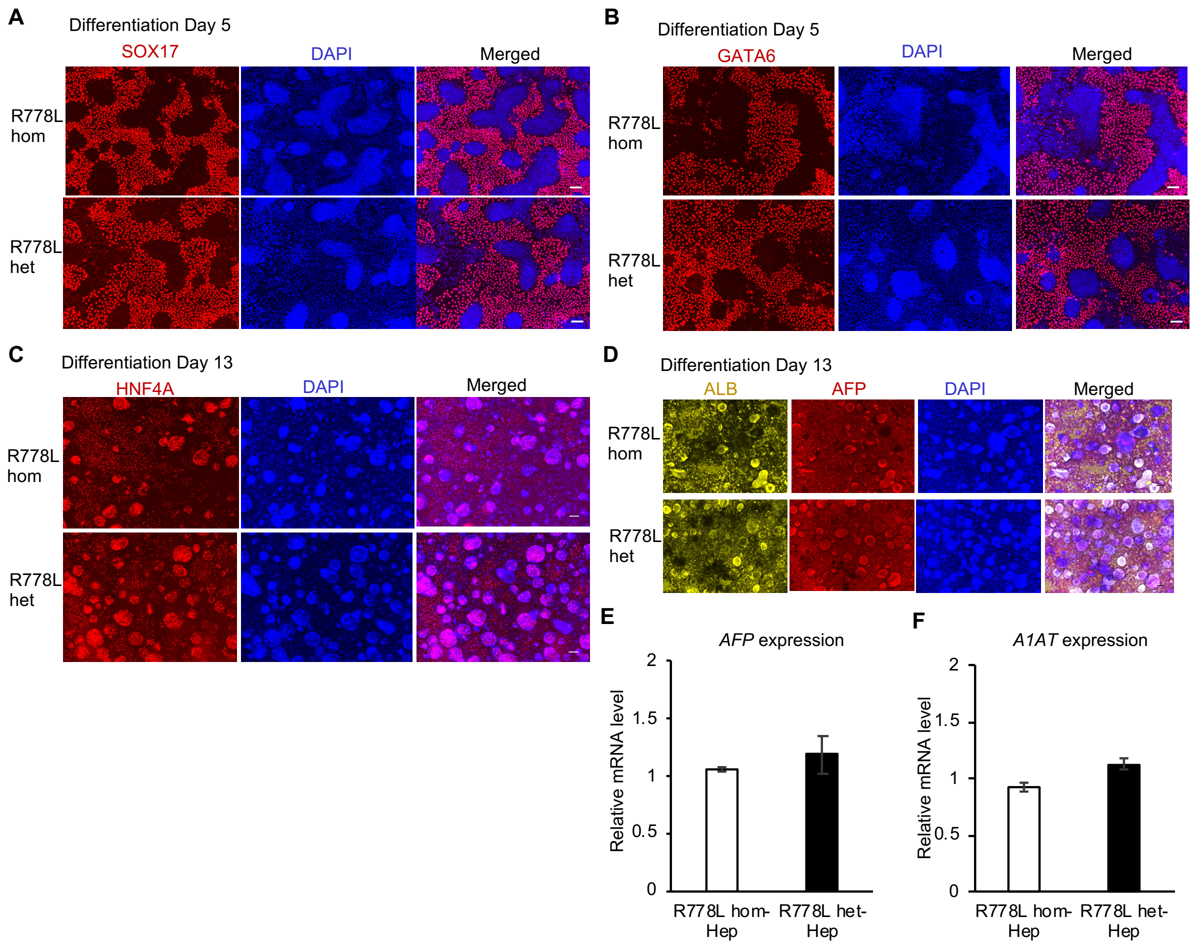

Supplementary Material, Figure S5. Hepatocyte differentiation ability of *ATP7B* R778L-heterozygous iPSCs gene-corrected from R778L-homozygous iPSCs, related to Fig. 5. (A and B) Representative immunostaining images showing the expression of endoderm markers, SOX17 (A) and GATA6 (B), on differentiation day 5 of *ATP7B* R778L-heterozygous (R778Lhet) and *ATP7B* R778L-homozygous (R778Lhom) iPSC clones. Scale bar=100 μm. **(C and D)** Representative immunostaining images showing the expression of hepatocyte markers HNF4A (C), AFP and ALB (D) on differentiation day 13 of *ATP7B* R778Lhet and R778Lhom iPSC clones. Scale bar=100 μm. **(E and F)** The gene expression of hepatic markers *AFP* (E) and *A1AT* (F) detected by RT-qPCR in hepatocytes derived from *ATP7B* R778Lhet and R778Lhom iPSC clones (n=3). Bar graphs show the mean ± SEM.

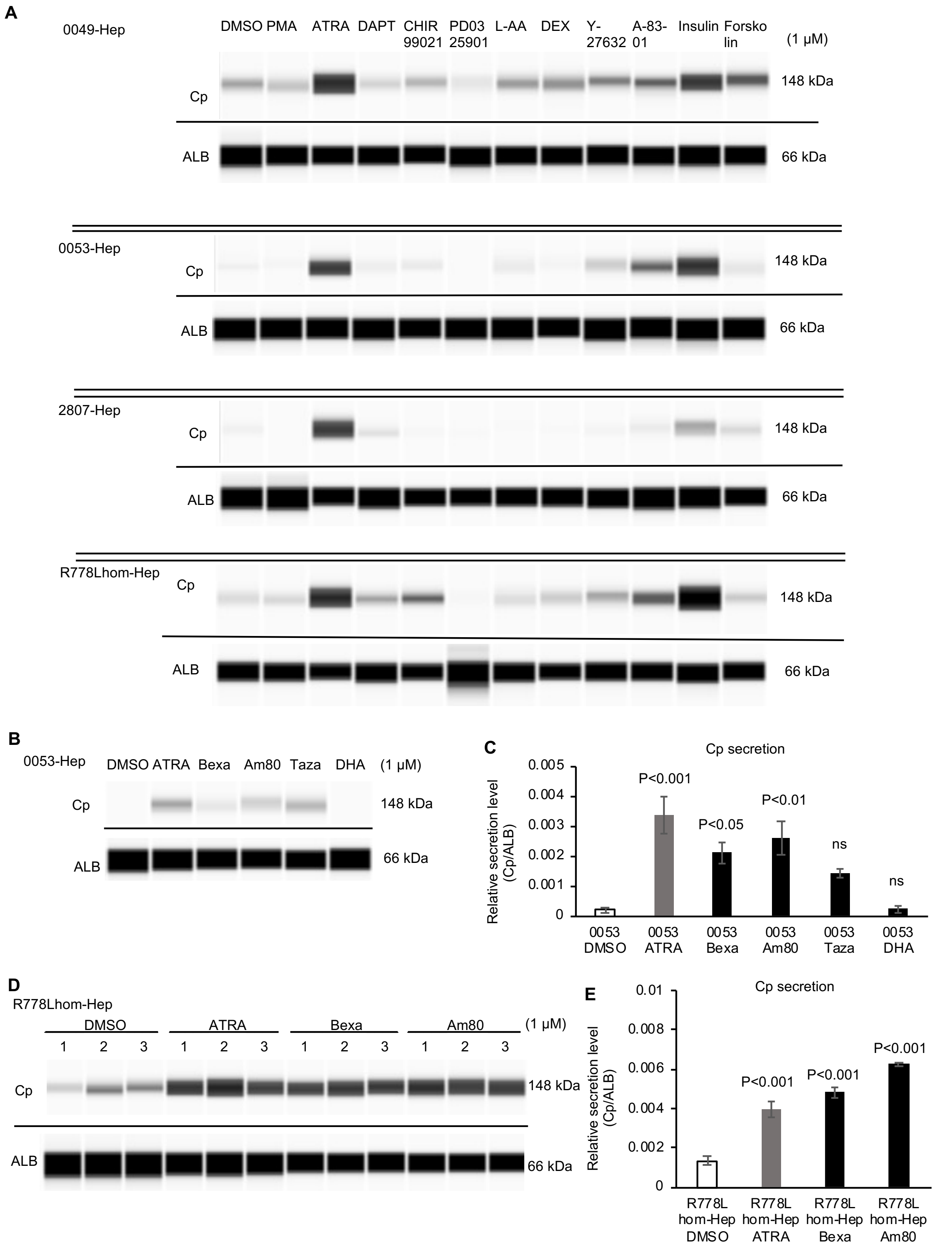
A
0049-Hep
DMSO PMA ATRA DAPT CHIR 99021 PD03 25901 L-AA DEX Y-27632 A-83-01 Insulin Forsko lin (1 μM)
Cp
ALB
148 kDa
66 kDa
0053-Hep
2807-Hep
R778Lhom-Hep
B
0053-Hep
DMSO ATRA Bexa Am80 Taza DHA (1 μM)
C
Cp secretion
Relative secretion level (Cp/ALB)
P<0.001
P<0.05
P<0.01
ns
0053 DMSO
0053 ATRA
0053 Bexa
0053 Am80
0053 Taza
0053 DHA
D
R778Lhom-Hep
DMSO ATRA Bexa Am80 (1 μM)
1 2 3
E
Cp secretion
R778L hom-Hep DMSO
R778L hom-Hep ATRA
R778L hom-Hep Bexa
R778L hom-Hep Am80

Supplementary Material, Figure S6. CP secretion-based drug screening identified ATRA to increase CP secretion in WD-iPSC-derived hepatocytes, related to Fig. 7. (A) Representative images of Western blot data of Cp and ALB proteins secreted by 0049-Hep, 0053-Hep, 2807-Hep, and R778L homozygous (R778Lhom)-Hep treated with the indicated drugs for four days on differentiation day 17. **(B)** Representative images of Western blots showing the Cp secretion of 0053-Hep on day 17 after treatment with indicated RAR/RXR agonists. **(C)** Quantified data of secreted CP protein after normalization with ALB protein in 0053-Hep following treatment with ATRA and the two indicated RXR and RAR agonists. Data are shown as the mean ± SEM (n=3). P-values were determined by Dunnett's test relative to DMSO-treated cells. **(D)** Representative images of Western blots showing CP and ALB secreted by R778L-homozygous iPSC-derived hepatocytes (R778Lhom-Hep) treated with ATRA or the two indicated retinoids on differentiation day 17. **(E)** Quantified data of the Cp secretion after normalization with ALB protein in R778Lhom-Hep on differentiation day 17 following treatment with ATRA or the two indicated retinoids (n=3). Bar graphs show the mean ± SEM. P-values were determined by Dunnett's test relative to DMSO-treated cells.

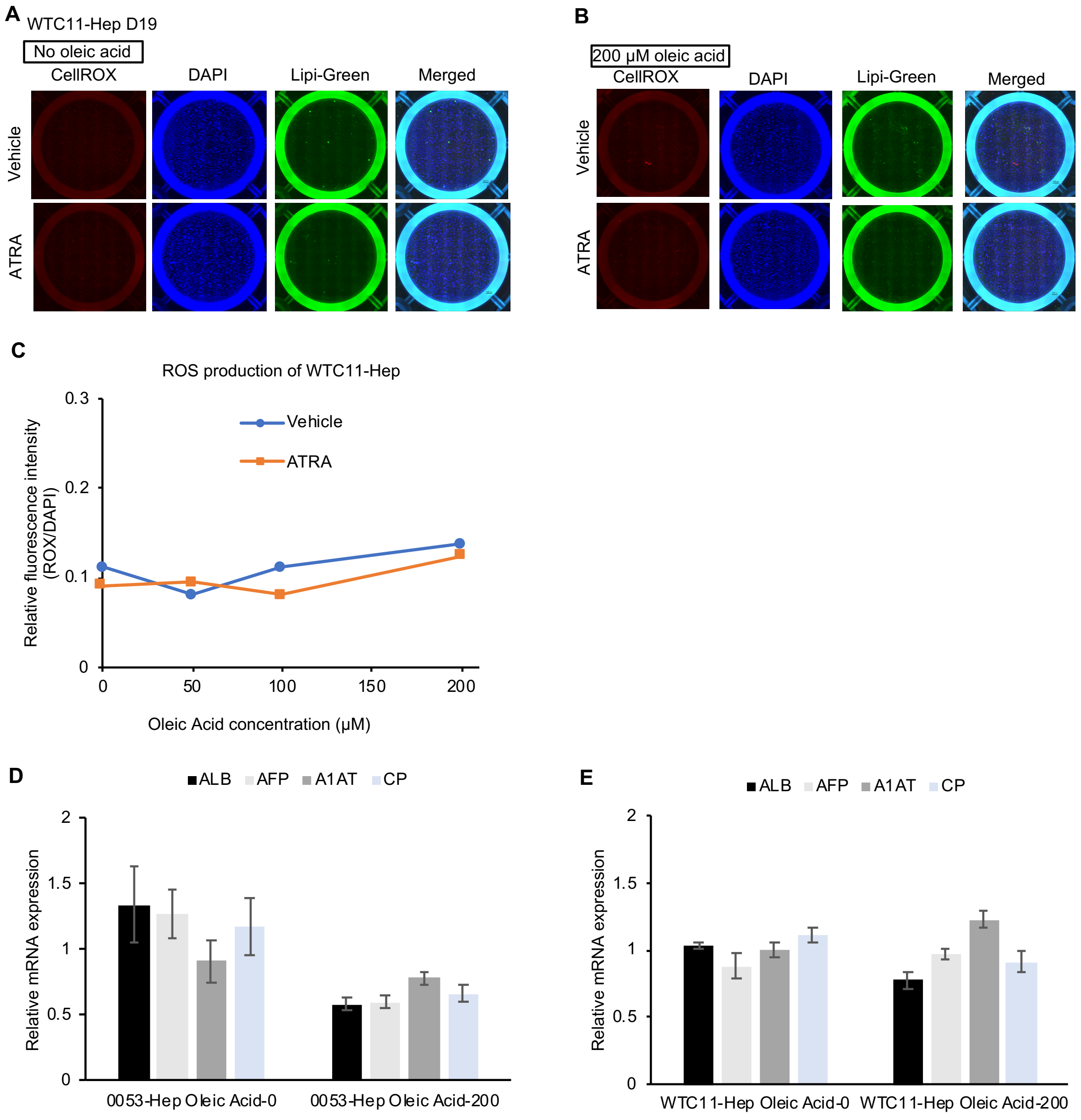

Supplementary Material, Figure S7. ROS production and lipid accumulation in WD-iPSC-derived hepatocytes, related to Fig. 8. (A and B) Tiled whole-well fluorescent images of CellROX, DAPI, and Lipi-Green in WTC11-derived hepatocytes on differentiation day 19 after oleic acid treatment at 0 µM (B) or 200 µM (C) for 8 days with or without ATRA for 4 days. **(C)** Quantified lipid accumulation at different concentrations of oleic acid with or without ATRA in WTC11-derived hepatocytes (day 19). **(D and E)** Gene expression of *ALB*, *AFP*, *A1AT,* and *CP* in HPS0053-derived hepatocytes (D) and WTC11-derived hepatocytes (E) treated with or without oleic acid (200 µM) for 6 days were analyzed by RT-qPCR on differentiation day 17 (n=3). Bar graphs show the mean ± SEM.

For Fig. 3D

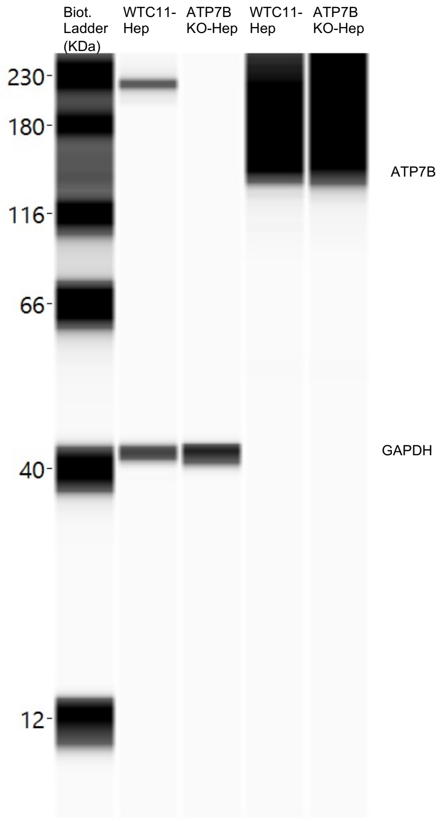

For Fig. 3I

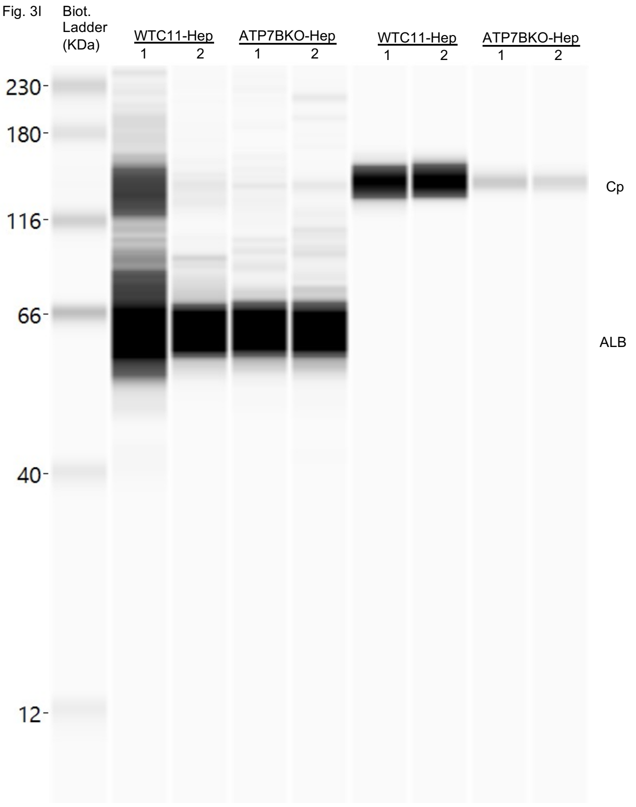

For Fig. 5D

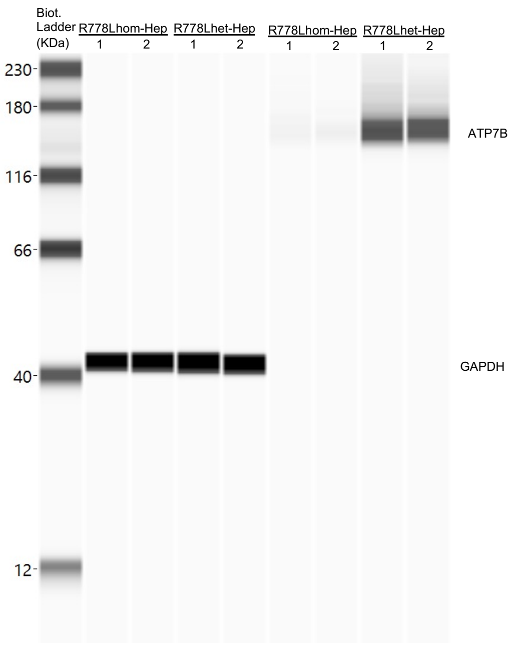

For Fig. 5G

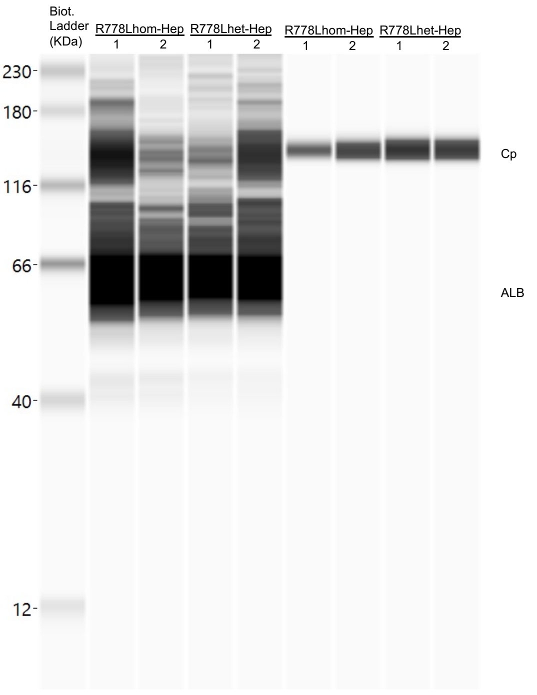

For Fig.7B

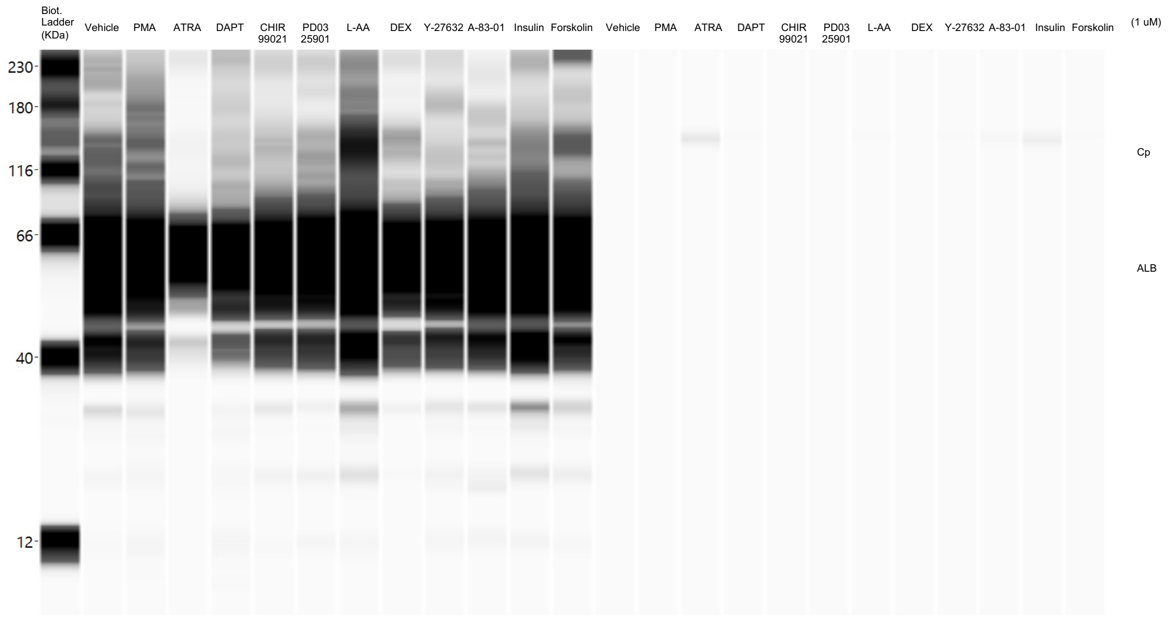

For Fig.7E

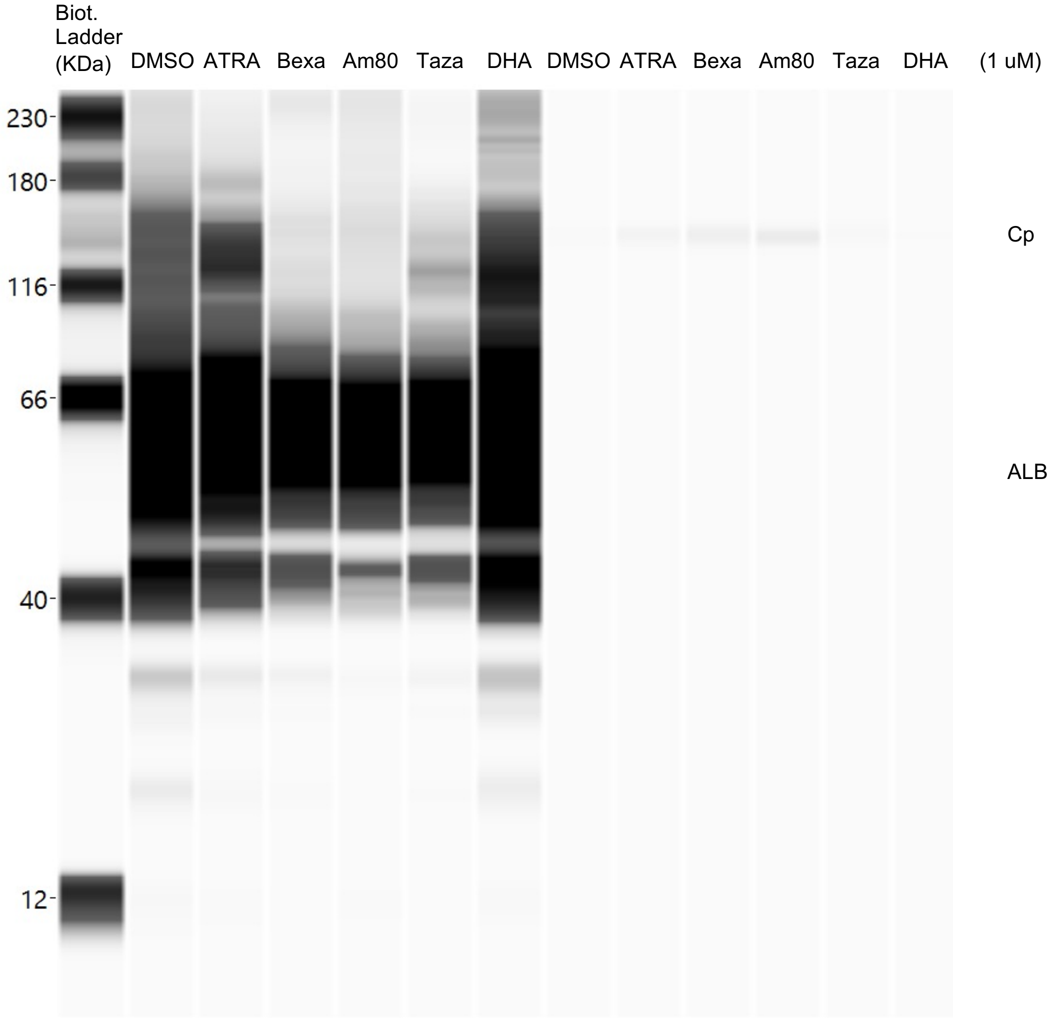

Supplementary Material, Figure S8. The entire gel images of the Simple Wes assay results for Fig. 2B, Fig 3D and I, Fig. 5D and G, and Fig 7B and E.

Supplementary Material, Table S1. Summary of information on the WD-patient-derived iPSC lines generated in this study, related to Fig. 1

iPSC cell line name	Gender	Age of patient at sampling (years)	Ethnicity	Disease	Origin	Exogenous genes
HPS2807	Female	40s	Japanese	Wilson's disease	Peripheral blood mononuclear cell	Oct3/4, Sox2, Klf4, L-Myc, Lin28, mp53DD, EBNA1
HPS0045	Female	Not disclosed	Japanese	Wilson's disease	Skin fibroblast	pMXs-Oct3/4, -Sox2, -Klf4, -c-Myc
HPS0049	Male	Not disclosed	Japanese	Wilson's disease	Skin fibroblast	pMXs-Oct3/4, -Sox2, -Klf4, -c-Myc
HPS0053	Female	Not disclosed	Japanese	Wilson's disease	Skin fibroblast	pMXs-Oct3/4, -Sox2, -Klf4, -c-Myc

Supplementary Material, Table S4. Summary of iPSC/ESC models derived from WD patients.

Cell type	Name (Alternative name)	DNA change*	Protein change	Zygosity	Mutation position in protein domain	Reference
iPSC	WD iPSC	c.2333G>T	p.R778L	Homozygous	TM (1-6)	Zhang et al., Human Molecular Genetics 2011, Vol. 20, No. 16.
iPSC	Not defined	c.2305A>G	p.M769V	Homozygous	TM (1-6)	Yi et al., Protein Cell 2012, 3(11): 855–863.
iPSC	Not defined	c.3207C>A	p.H1069Q	Homozygous	ATP binding	Parisi et al., Scientific Report 2018, 8:6247.
hESC	hESBGN-01 (BG01)	c.2333G>T	p.R778L	Homozygous	TM (1-6)	Kim et al., Cells 2020, 9, 872.
iPSC	ZZUNEUi003-A	c.2333G>T	p.R778L	Homozygous	TM (1-6)	Zhang et al., Stem Cell Research 2019, 41:101664.
iPSC	AKOSi003-A (F0101990-1)	c.2305A>G	p.M769V	Homozygous	TM (1-6)	Petters et al., Stem Cell Research 2020, 43:101708
iPSC	AKOSi002-A2 (GM05257-11)	c.3191A>C<br>c.3207C>A	p.E1064A<br>p.H1069Q	Compound heterozygous	ATP binding	Petters et al., Stem Cell Research 2020, 43:101708
iPSC	ZZUNEUi004-A	c.2975C>T	p. P992L	Homozygous	TM (1-6)	Zhang et al., Stem Cell Research 2020, 44:101741.
iPSC	ZJUi003-A (GZW-iPS)	c.180_181del	p.T61Sfs*16	Heterozygous	Cu binding(?)	Wang et al., Stem Cell Research 2020, 46:101873.
iPSC	ZZUNEUi005-A	c.2975C>T	p. P992L	Homozygous	TM (1-6)	Wei et al., Stem Cell Research 2020, 47:101909.
iPSC	ICGi020-A (ATP7bIL23f)<br>ICGi020-B (ATP7bIL24f)	c.3207C>A	p.H1069Q	Heterozygous	ATP binding	Malakhova et al., Stem Cell Research 2020, 47:101922.

iPSC	ICGi030-A (HF2)	c.3036dup<br>c.3207C>A	p.K1013fs<br>p.H1069Q	Compound heterozygous	TM (1-6)<br>ATP binding	Zhigalina et al., Stem Cell Research 2021, 57:102556.
iPSC	HPS0045 (HiPS-RIKEN-5A)	c.2333G>T,<br>c.2495A>G<br>c.2855G>A	p.R778L,<br>p.K832R<br>p.R952K	Heterozygous	TM (1-6)	First reported in this study.
iPSC	HPS0049 (HiPS-RIKEN-7A)	c.3104G>T<br>c.3784G>T	p.G1035V<br>p.V1262F	Heterozygous	TM (1-6)<br>ATP binding	First reported in this study.
iPSC	HPS0053 (HiPS-RIKEN-9A)	c.3104G>T<br>c.3784G>T	p.G1035V<br>p.V1262F	Heterozygous	TM (1-6)<br>ATP binding	First reported in this study.
iPSC	HPS2807	c.1216T>G,<br>c.1366G>C<br>c.2495A>G,<br>c.2855G>A<br>c.2975C>T,<br>c.3029A>C	p.S406A,<br>p.V456L<br>p.K832R,<br>p.R952K<br>p.P992L,<br>p.K1010T	Heterozygous	Cu binding<br>TM (1-6)<br>ATP binding	First reported in this study.
iPSC	R778Lhet	c.2333G>T	p.R778L	Heterozygous	TM (1-6)	First reported in this study.<br>(Corrected with Heterozygous the R778L homozygous mutation in Zhang et al., 2011)
Gene-edited iPSC	WTC11 exon1 deletion	c.159_165del<br>c.164_267del	Exon1 deletion	Compound heterozygous	Translation start site	First reported in this study.<br>(Modified WTC11 iPS cells)
Gene-edited iPSC	WTC11<br>R778L homozygous	c.2333G>T	p.R778L	Homozygous	TM (1-6)	First reported in this study.<br>(Modified WTC11 iPS cells)

Supplementary Material, Table S5. List of primers used for ATP7B genome DNA and transcript amplification and sequencing.

Name	Forward/Reverse primer (5′−3′)
ATP7B cDNA Primer 1	F: Tcctctcccgggactttaac/R: cagttggagggtgaccacat
ATP7B cDNA Primer 2	F: Agagcaaagtggctccctta/R: accaacacggagagaacacc
ATP7B cDNA Primer 3	F: aagtccccacaatcaaccag/R: ggcaatgaacacaaagagca
ATP7B cDNA Primer 4	F: Ctctggtcatcctggtggtt/R: ggtgccagtcttgtcaaaca
ATP7B cDNA Primer 5	F: aaccccaacaagcacatctc/R: atggcgactttcttcccttt
ATP7B cDNA Primer 6	F: Aatcgcagacgctgtcaag/R: tcagcttgtggtgagtggag
ATP7B cDNA Primer 7	F: gggacaagtggtctctgctc/R: ccgagtacggactctcaggt
ATP7B cDNA Primer 8	F: Tgcttcctggctcacacat/R: tcacagctgacagacactgct
ATP7B cDNA Primer 9	F: aggggcttcgttttcagtct/R: aacaacattccccaagcaag
ATP7B cDNA Primer 10	F: Tgattgacagaacccctttgg/R: tttttagaggaatgacaggaacttt
ATP7B gDNA 406	F: gaagggagtgggacagatca/R: catgcaaggaaagtttgcag
ATP7B gDNA 456	F: gatggctgagggacaaggta/R: gggcgttcatctcttaccag
ATP7B gDNA 778	F: gagacacaggtcagccaaca/R: cctgaaggccaggtttcttt
ATP7B gDNA 832	F: tcagcagctgcacgataaat/R: ttacagtgcttccgggtttc
ATP7B gDNA 952	F: tgtgaagagttctgggaaatca/R: cctgcagaaggagagtgactg
ATP7B gDNA 1035	F: tgcaggtgtcttgtttcctg/R: ggccctctaagtggttttcc
ATP7B gDNA 1262	F: caggagccagggataaactg/R: ctgatggagaggagcacaca

Supplementary Material, Table S6. List of ddPCR probes and primers used for genome editing in this study.

ATP7B deletion mutagenesis

Name	**Forward/Reverse primer (5′−3′)**
ATP7B KO ddPCR primer	F: CTCTCCCGGGACTTTAAC<br>/R: AAACTCACTTTCCGACTG
ATP7B KO Ref FAM probe	TGAGCCAGATCAGAGAAGAA
ATP7B KO NHEJ HEX probe	CTCTCCTGCTCAGGCATC

ATP7B R778L point mutagenesis or correction

Name	**Forward/Reverse primer (5′−3′)**
ATP7B R778L ddPCR primer	F: tgcttatgtttattctctggtcatc<br>/R: cctgaagctgctgttacctt
ATP7B R778L Ref FAM probe	tggtggttgctgtggct
ATP7B R778L HDR FAM probe	TGGGCCTGTGGCTG
ATP7B R778L+SM HDR FAM probe	TGGGCCTCTGGCTG
ATP7B R778L NHEJ HEX probe	CTGGGCCGGTGGCTG

Supplementary Material, Table S7. The list of Taqman probes used in this study.

**Gene name**	**Assay ID**
AFP	Hs01040598_m1
ALB	Hs00609411_m1
SOX17	Hs00751752_s1
ATP7B	Hs01075310_m1
CP	Hs00236810_m1
SERPINA1	Hs00165475_m1
HNF4A	Hs00230853_m1
DLK1	Hs00171584_m1

Supplementary Material, Table S8. The list of primary antibodies used in this study.

Primary Abs	Host species	Dilutions	SOURCE	IDENTIFIER
SOX17	Goat	1:500	R&D systems	Cat#AF1924
GATA6	Goat	1:500	R&D systems	Cat#AF1700
CP	Rabbit	1:300	Cell Signaling Technology	Cat#98971
Albumin	Goat	1:300	Bethyl	Cat#A80-229A
Alpha-Fetoprotein	Mouse	1:300	R&D systems	Cat#MAB1368
HNF4A	Mouse	1:300	R&D systems	Cat#MAB4605-SP
GAPDH	Mouse	1:500	R&D systems	Cat#MAB5718
NANOG	Rabbit	1:750	Reprocell	Cat#RCAB004P-F
OCT-3/4	Goat	1:500	R&D systems	Cat#AF1759-SP
OCT-3/4	Mouse	1:300	Santa Cruz	Cat#sc-5279
KLF4	Rabbit	1:750	MBL	Cat#PM057
SOX2	Mouse	1:500	R&D systems	Cat#MAB2018
SOX2	Goat	1:500	R&D systems	Cat#AF2018
Alpha-SMA	Mouse	1:750	R&D systems	Cat#MAB1420
p230	Mouse	1:400	BD	Cat#611280
ATP7B	Rabbit	1:250	Novus Biologicals	Cat#NB100-360
ATP7B	Rabbit	1:5000 (Wes) 1:250 (ICC)	Abcam	Cat#ab124973

Supplementary Material, Table S9. The list of secondary antibodies used in this study.

**Secondary Abs**	**Dilution**	**SOURCE**	**IDENTIFIER**
Donkey anti-Goat IgG (H+L) Highly Cross-Adsorbed Secondary Antibody, Alexa Fluor Plus 647	1:1000	Thermo Fisher Scientific	Cat#A32849
Alexa Fluor 555-conjugated goat anti-rabbit IgG	1:1000	Thermo Fisher Scientific	Cat#A21428
Donkey anti-Goat IgG (H+L) Highly Cross-Adsorbed Secondary Antibody, Alexa Fluor Plus 555	1:1000	Thermo Fisher Scientific	Cat#A32816
Donkey anti-Mouse IgG (H+L) Highly Cross-Adsorbed Secondary Antibody, Alexa Fluor 555	1:1000	Thermo Fisher Scientific	Cat#A-31570
DyLight 649 Donkey anti-rabbit IgG	1:1000	Biolegend	Cat#406406

Supplementary Material, Table S10. The list of chemicals used in this study.

**Chemical name**	**SOURCE**	**IDENTIFIER**
PMA	Wako	Cat#183882
ATRA	Sigma-Aldrich	Cat#R2625
DAPT	Calbiochem	Cat#565784
CHIR99021	Wako	Cat#034-23103
PD0325901	Wako	Cat#162-25291
L(+)-Ascorbic Acid (L-AA)	Wako	Cat#012-04802
Dexmethathone	Nacalai Tesque	Cat#11107-64
Y-27632	Wako	Cat#034-24024
A8301	Sigma-Aldrich	Cat#SML0788
Insulin	Nacalai Tesque	Cat#12878-86
Forskolin	Nacalai Tesque	Cat#16384-84
cis-4,7,10,13,16,19-Docosahexaenoic acid	Sigma-Aldrich	Cat#D2534
Bexarotene	Sigma-Aldrich	Cat#SML0282
Am80	Wako	Cat#017-16621
Tazarotene	Wako	Cat#517-87741

Supplementary Material, Table S11. The list of reagents used in this study.

REAGENT or RESOURCE	SOURCE	IDENTIFIER
iMatrix-511, silk (Laminin-511)	Nippi	Cat# 892021
StemFit AK02N medium	Ajinomoto	Cat# AK02N
CultureSure Y-27632	Wako	Cat#034-24024
Chromosome Analysis Suite	Thermo Fisher Scientific	
FastGene RNA premium kit	Nippon	Cat#FG-81250
ReverTra Ace qPCR RT kit	Toyobo	
Tks Gflex DNA Polymerase	Takara	Cat#R060A
FastGene Gel/PCR extraction kit	Nippon	Cat#FG-91202
FastGene Plasmid Mini Kit	Nippon	Cat#FG-90502
THUNDERBIRD Probe qPCR Mix	Toyobo	Cat#QPS-101X5
StemSure DMEM	Wako	Cat#197-16275
L-alanyl-L-Glutamine solution	Nacalai Tesque	Cat#04260-64
0.5mol/l-EDTA Solution (pH 8.0)	Nacalai Tesque	Cat#06894-14
50 mM Monothioglycerol Solution	Wako	Cat#195-15791
MEM non-essential amino acids	Nacalai Tesque	Cat#06344-56
DMSO solution	Sigma-Aldrich	Cat#D2650 100ML
Penicillin (50U/ml 3uM)/Streptomycin (50ug/ml)	Nacalai Tesque	Cat#26252-94
DMEM/Ham's F12	Nacalai Tesque	Cat#11581-15
StemSure serum replacement	Wako	Cat#191-18375
High efficiency DH5ALPHA Competent Cell	GMbiolab	Cat#DH01-100
LB Broth, Miller	Nacalai Tesque	Cat#20068-75
LB Agar, Miller	Nacalai Tesque	Cat#20069-65

Mighty Cloning Reagent Set (Blunt End)	Takara	Cat# 6027
B27 minus insulin	Thermo Fisher Scientific	Cat#A1895601
Recombinant human Oncostatin M	Wako	Cat#153-02101
Recombinant human Hepatocyte Growth Factor	Wako	Cat#082-08721
Activin A Solution, Human, recombinant	Wako	Cat#010-27623
Hepatocyte Basal Medium	Lonza	Cat#CC-3199
SDS-PAGE Sample Buffer Solution without 2-ME(2x) for SDS-PAGE	Nacalai Tesque	Cat#30567-12
Dithiothreitol (DTT)	Nacalai Tesque	Cat#14130-41
Protease inhibitor cocktail solution	Nacalai Tesque	Cat#04080-11
Pierce 660 nm Protein Assay Reagent	Thermo fisher	Cat#22662
Jess/Wes 12-230 kDa 8X25 capillaries cartridges separation module	ProteinSimple	Cat#SM-W004
Anti-Rabbit Detection Module for Jess, Wes, Peggy Sue or Sally Sue	ProteinSimple	Cat#DM-001
Anti-Mouse Detection Module for Jess, Wes, Peggy Sue or Sally Sue	ProteinSimple	Cat#DM-002
Anti-Goat Detection Module for Jess, Wes, Peggy Sue or Sally Sue	ProteinSimple	Cat#DM-003
STEM-CELLBANKER GMP Grade	Takara Bio	Cat# CB045
CellROX Deep Red Reagent	Thermo Fisher Scientific	Cat#C10491
Oleic acid	Sigma-Aldrich	Cat#O1383-1G
Lipi-Green reagent	DojinDo	Cat#LD02
96 well-V-plate	EZ-BindShut SP	Cat#4420-800SP
